# Trends in socioeconomic inequalities in behavioural non-communicable disease risk factors: analysis of repeated cross-sectional health surveys in England between 2003 and 2019

**DOI:** 10.1186/s12889-023-16275-6

**Published:** 2023-07-28

**Authors:** Fatai Ogunlayi, Paul C. Coleman, Linda Ng Fat, Jennifer S. Mindell, Oyinlola Oyebode

**Affiliations:** 1 University of Warwick, Warwick Medical School, Coventry, CV4 7AL UK; 2 Centre for Food Policy, City University of London, London, EC1V 0HB UK; 3 Health and Social Surveys Group, Research Department of Epidemiology and Public Health, University College London (UCL), 1-19 Torrington Place, London, WC1E 7HB UK; 4 Wolfson Institute of Population Health, Queen Mary University of London, London, EC1M 6BQ UK

**Keywords:** Health inequalities, Healthcare disparities, Health policy, Public health, England

## Abstract

**Background:**

Previous studies have shown that those in lower socioeconomic positions (SEPs) generally have higher levels of behavioural non-communicable disease (NCD) risk factors. However, there are limited studies examining recent trends in inequalities. This study examined trends in socioeconomic inequalities in NCD behavioural risk factors and their co-occurrence in England from 2003–19.

**Methods:**

This time-trend analysis of repeated cross-sectional data from the Health Survey for England examined the relative index of inequalities (RII) and slope index of inequalities (SII) in four NCD behavioural risk factors: smoking; drinking above recommended limits; insufficient fruit and vegetables consumption; and physical inactivity.

**Findings:**

Prevalence of risk factors has reduced over time, however, this has not been consistent across SEPs. Absolute and relative inequalities increased for physical inactivity; relative inequalities also increased for smoking; for insufficient fruit and vegetable consumption, the trends in inequalities depended on SEPs measure. Those in lower SEPs experienced persistent socioeconomic inequalities and clustering of behavioural risk factors. In contrast, those in higher SEPs had higher prevalence of excessive alcohol consumption; this inequality widened over the study period.

**Interpretation:**

Inequalities in smoking and physical inactivity are persisting or widening. The pattern of higher drinking in higher SEPs obscure the fact that the greatest burden of alcohol-related harm falls on lower SEPs. Policy attention is required to tackle increasing inequalities in smoking prevalence, low fruit and vegetable consumption and physical inactivity, and to reduce alcohol harm.

## Summary boxes

Section 1: What is already known on this topic

• Those in lower socio-economic positions (SEPs) have generally higher levels of behavioural non-communicable disease (NCD) risk factors than those in higher SEPs.

• Behavioural NCD risk factors tend to co-occur.

• Whilst levels of some behavioural risk factors have been declining over the past decade it is unclear how this has occurred across SEPs and whether inequalities are widening or narrowing over time.

Section 2: What this study adds

• From 2003–2019, prevalence of smoking, excessive alcohol consumption, low fruit and vegetable consumption, and physical inactivity declined, however social-economic inequalities widened for smoking and physical inactivity.

• On both relative and absolute scales, social-economic inequalities for low fruit and vegetable consumption narrowed across neighbourhood deprivation and income for women but widened across educational level for men, suggesting differential effects by sex and SEPs.

• Those in lower SEPs had a higher prevalence of having two or more behavioural risk factors; this remained stable over the time-period.

## Background

Non-communicable diseases (NCDs) cause an estimated 41 milliondeaths each year, 71% of all deaths globally [1]. Approximately 7.6 million people in the UK are estimated to be living with cardiovascular disease (CVD) [2] and 2.9 million with cancer [3]. This places a substantial demand on health services and society: prevention is crucial to reducing the morbidity and mortality associated with these diseases.

Four key modifiable behaviours are known to increase NCD risk, namely: tobacco use, unhealthy diet, physical inactivity and harmful use of alcohol [1]. There is also growing evidence suggesting that behavioural risk-factors often co-occur or cluster in individuals [4]. Where these behaviours co-occur, they are synergistic rather than additive (i.e., combination of risks may be greater than would be expected from adding up the individual risks alone) [4].

The risk of NCD mortality and morbidity is generally highest for those in the most deprived socioeconomic positions (SEPs) [5]. In addition, although the relationship is complex, generally more deprived groups have higher rates of behavioural risk factors [6]. For some populations, differing prevalence of behavioural risk-factors by SEPs has been found to explain most of the relationships between SEPs and NCD mortality. For example smoking and alcohol use explained much of the educational inequality in CVD in a Dutch cohort [7]. Further, although public health interventions have aimed to reduce the prevalence of behavioural risk-factors, some interventions are potentially less effective for the most deprived population groups [8, 9]. Perhaps as a consequence of this, changes in prevalence of some NCDs has been uneven. For example, an analysis of coronary heart disease mortality in England from 1982–2006 found steeper falls in mortality rates in the least deprived areas so that relative inequality increased significantly, although absolute inequality declined [10].

Socioeconomic position is “an aggregate concept that includes both resource-based and prestige-based measures” [11]. Having a low SEP can mean being deprived of material resources, having limited opportunities, low social status, and exposure to an adverse social and physical environment at home and at work. Four measures of SEPs have often been used to examine the association with health: educational attainment, employment status, income level, and neighbourhood deprivation [12]. These measures each relate to a different aspect of an individual’s SEP, and may be associated with NCD risk through different, although overlapping, pathways. For this reason, each measure may have differing associations with NCD risk. For example, in a study of a New Zealand population, CVD risk-factors were more strongly associated with area-based deprivation and income inequality than with occupation or education [13].

The aim of this study was to examine the national trends in socioeconomic inequalities in four behavioural NCD risk factors and their co-occurrence in England, using the nationally representative Health Survey for England (HSE) data. Additionally, this study examines whether there are differences depending on the SEPs measure used.

## Methods

### Survey design

This study used data collected in the HSE from 2003, when the ability to account for non-response weighting was introduced, to 2019 for the adult population (aged 16 years and over). HSE is a series of annual surveys of people living in private households in England. The detailed methodology of the survey has been described elsewhere [14]. In 2005, there was a boost sample of participants aged 65 + , but to retain national representativeness and ensure comparable year on year analyses, only the core sample has been used. Interview weightings were applied in this study as all risk factors were derived from the interview stage of the survey. Household response rates to health examination surveys have steadily decreased over time in England and other countries [15].

### Patient and public involvement

Patients and the public were not involved in this secondary analysis. Public sector stakeholders are included in the HSE Steering Group that considers topics for inclusion each year.

### Data collection and definitions

#### CVD risk factors measurement

Data on four behavioural risk factors were self-reported using standard questions [14] and were subsequently dichotomised as follows: (i) being a current cigarette smoker, (ii) drinking more than the UK previous recommended daily guidelines, based on the heaviest drinking day in the past week (4 units/d for men, 3 units/d for women), (iii) consuming fewer than the recommended five portions of fruit and vegetables per day and (iv) being physically inactive (spending < 30 min per week in moderate-to-vigorous intensity physical activity). Availability of each risk factor by survey year is presented in Table 1.

**Table 1:** Behavioural risk factors and years of data available

Risk factors	Details	Comparable years of data used	Total participants (aged 16 + with no missing data)
Alcohol	Drinking more than sensible daily alcohol intake defined by consumption of < = 3 units of alcohol for women and < = 4 units of alcohol for men	2007–2019	108,200
Smoking	Current cigarette smoker	2003–2019	154,121
Fruit & Vegetable	Consuming fewer than the recommended five portions of fruit and vegetables per day	2003–2011, 2013, 2015–2018	127,936
Physical inactivity	Being physically inactive by spending less than 30 min per week in moderate-to-vigorous intensity physical activity	2003, 2004, 2006, 2008, 2012, 2016	65,178
Behavioural Multiple risk factors	Combining Alcohol, Smoking, and Fruit & Vegetables	2007–2011, 2013, 2015–2018	84,646

#### Multiple risk factors

Physical inactivity was excluded from analyses of co-occurrence of multiple behavioural risk-factors (MRF) because its inclusion would have limited the analyses of multiple risk factors to only two time points when all four are available: 2008 and 2016.

The remaining three behavioural risk factors (excessive alcohol intake, smoking, and insufficient fruit & vegetable consumption) were summed at the individual participant level, with individuals classified as having 0–3 behavioural risk-factors. Only the years where all three behavioural risk factors were collected have been included in the MRF analyses (2007–2011; 2013; 2015–2018).

#### Socioeconomic positions

Individual and area-level factors can both contribute to health outcomes with complex relationship between them. Examining both types of measures provides a more comprehensive understanding of socioeconomic inequalities and could inform the development of targeted policies and interventions that address multiple levels of influence.

Socioeconomic position was measured using four indicators. Area deprivation related to the individual’s home address, as measured by the index of multiple deprivation (IMD) 2015 (grouped into quintiles). The remaining three was collected via self-report at the main interview; highest educational attainment level (grouped into degree or equivalent, below degree, and no qualification); equivalised net disposable household income (adjusted for household composition and grouped into quintiles); and occupational status (grouped into managerial/professional, intermediate, manual and other).

### Statistics analyses

We maximised the sample by using all available cases, resulting in differing sample sizes across each variable, predominately driven by inconsistency in data collection over the study period (Table 1). The maximum sample size was for smoking (*N* = 154,121), followed by fruit & vegetable consumption (*N* = 127,936), alcohol (*N* = 108,200), behavioural MRF (*N* = 84,646) and physical inactivity (*N* = 65,178).

Direct age standardisation was carried out for prevalence of each risk factor using the population estimates for England for age groups 16–24, 25–34, 35–44, 45–54, 55–64, 65–74 and 75 + , derived from mid-year 2019.

The relative index of inequality (RII, measures relative change in inequality) and slope index of inequality (SII, measures absolute change in inequality) are the recommended measures to use when measuring change in inequality over time as they take into account the whole socioeconomic distribution and changes in population share of socioeconomic groups [16]. Reporting both measures is important to enable understanding of inequalities in NCD risk factors and to inform targeted policy interventions aimed at reducing both relative and absolute inequalities. Discrepancies in RII and SII trends would highlight the need to consider the underlying factors that are driving these inequalities.

To calculate RII and SII for each survey year, categories of each SEP at each survey were transformed into a summary measure referred to as a ‘ridit’ score, weighted to reflect the proportion of the sample at each category. Detailed description of how to calculate the ridit score have been described elsewhere [17]. The ridit scores were then included in linear probability models. A generalised linear model, with a logarithmic link function was used to estimate the RIIs and with an identity link function to estimate SIIs [16]. Due to well-documented convergence problems with log-binomial regressions, a log-Gaussian regression was used as an alternative as suggested in the literature [18]. The models were stratified by sex and adjusted for age. Missing data were excluded from analyses.

To estimate the trends in RII and SII over the survey years, the year variable was converted into a continuous variable in order to account for the different time periods between surveys, as recommended in the literature [19]. An interaction term between the derived ridit score for each socioeconomic variable and derived continuous year variable was included in the generalised linear models.

Analyses were conducted using Stata v16 and have taken into account the HSE’s clustered, stratified design and non-response weighting using Stata’s complex survey ‘svy’ prefix command. Strata with a single sampling unit were treated as certainty units.

## Results

### Descriptive analyses of the study population

Characteristics of the study population are shown in Table 2 (*N* = 155,226 adults aged 16 +). Between 2003 and 2019, the proportion of participants with a high education level (degree or equivalent) increased considerably from 19 to 30% for men and from 15 to 30% for women. There was a smaller increase in the proportion of participants with high occupational status (managerial or professional, and intermediate) from 53 to 57% for men and from 51 to 59% for women. For most variables, missing data was non-existent or small (< 1–3%), with the exception of income where missing data ranged from 15%-24%.

**Table 2:** Characteristics of study population stratified by sex

Variables	2003	2004	2005	2006	2007	2008	2009	2010	2011	2012	2013	2014	2015	2016	2017	2018	2019
Men Total, n	6602	2879	4629	6324	3070	6759	2108	3702	3822	3680	3925	3588	3578	3552	3536	3669	3674
Age group																	
16–24 (%)	14.5	14.9	15.2	15.2	15.2	15.5	15.7	15.5	15.1	14.8	14.6	14.8	14.1	14.4	13.8	13.9	13.7
25–34 (%)	17.7	17.2	16.9	16.5	16.5	16.6	16.6	16.8	16.9	17.1	17.0	17.1	17.0	16.9	17.0	16.8	16.6
35–44 (%)	19.7	19.8	19.6	19.8	19.6	19.3	19.0	18.1	18.1	17.7	17.3	16.9	16.5	16.3	16.2	16.2	16.2
45–54 (%)	16.5	16.3	16.3	16.4	16.5	16.5	16.7	17.3	17.3	17.6	17.7	17.8	17.9	17.8	17.7	17.5	17.2
55–64 (%)	14.5	14.7	14.7	14.8	14.9	14.8	14.7	14.6	14.6	14.7	14.4	14.1	14.3	14.3	14.6	14.9	15.2
65–74 (%)	10.1	10.1	10.1	10.1	10.0	9.9	10.0	10.3	10.3	10.5	11.1	11.3	11.8	11.9	12.1	12.1	12.1
75 + (%)	7.0	7.1	7.2	7.2	7.3	7.4	7.4	7.6	7.7	7.6	7.9	8.0	8.3	8.3	8.5	8.7	9.0
Ethnicity																	
White (%)	90.3	91.6	90.1	88.7	87.9	88.2	88.7	86.8	87.4	87	86.3	86.9	86.9	85.5	86	84.2	82.7
Black (%)	2.2	2.6	1.4	3.0	2.7	2.3	2.5	3.3	2.7	2.3	2.5	2.3	2.7	3.6	2.6	2.8	3.1
Asian (%)	4.9	4.3	5.9	6.3	6.6	6.8	5.8	6.6	7.6	8.1	8.2	7.8	7.0	7.7	8.6	9.8	10.6
Mixed (%)	0.7	0.5	1.2	0.8	1.0	1.2	1.8	1.3	1.2	1.6	1.6	1.4	2.4	1.8	1.5	1.6	2.1
Others (%)	1.7	0.9	0.8	1.0	1.3	0.9	0.7	1.5	0.6	0.6	1.0	1.0	0.9	1.0	0.8	1.0	1.1
Missing/Unknown (%)	0.3	0.2	0.5	0.3	0.5	0.6	0.5	0.4	0.5	0.4	0.3	0.7	0.2	0.4	0.4	0.5	0.4
IMD Deprivation																	
1—Least deprived (%)	21.7	22.9	21.1	19.2	22.1	21.4	20.1	21.9	19.8	20.6	19.6	21.8	20.1	18.7	19.4	18.3	19.1
2 (%)	20.0	22.5	21.5	20.5	19.4	19.7	20.8	18.6	20.9	21.1	21.3	19.1	21.0	18.3	21.3	21.1	19.3
3 (%)	20.0	17.8	19.8	22.0	21.8	20.0	21.8	20.2	20.7	20.4	21.5	19.6	20.4	22.3	20.8	21.9	20.0
4 (%)	20.4	20.8	20.9	20.1	19.1	20.1	20.3	19.8	19.3	19.3	19.1	20.5	19	20.4	19.6	20.9	21.3
5—Most deprived (%)	17.8	15.9	16.6	18.2	17.6	18.8	16.9	19.5	19.4	18.6	18.5	19.0	19.4	20.3	19.0	17.8	20.1
Education																	
Degree or equivalent (%)	18.5	21.2	20.9	21.7	22.2	21.8	24.2	23.8	24.4	26.2	26.2	26.8	27.7	29.0	29.6	28.9	30.0
Below degree (%)	58.0	54.2	55.0	55.3	53.0	55.8	54.4	56.7	55.2	54.3	52.9	52.7	52.8	50.9	51.6	52.3	50.4
No qualification (%)	23.1	24.1	23.4	22.5	24.1	21.8	21.0	19.0	19.7	19.0	20.4	19.7	19.3	19.6	18.2	18.2	18.9
Unknown (%)	0.3	0.4	0.6	0.5	0.8	0.6	0.4	0.5	0.6	0.5	0.5	0.8	0.2	0.4	0.6	0.7	0.7
Occupational status																	
Managerial (%)	35.2	37.2	35.2	36.3	36.1	36.0	36.7	36.1	36.1	35.4	36.1	36.4	37.4	36.0	37.4	37.8	37
Intermediate (%)	18.0	17.7	18.0	18.0	17.8	18.2	18.4	18.1	18.7	20.3	19.5	18.6	19.2	19.8	19.3	18.8	19.8
Manual (%)	43.1	40.2	41	40.1	40.3	39.5	39.6	38.5	37.7	36.8	37.3	37.7	37.2	37	35.9	35.4	36.4
Other (%)	3.4	4.6	5.4	5.1	5.3	5.8	4.8	5.8	5.6	6.2	5.8	5.6	4.7	5.7	5.9	5.8	5.1
Unknown (%)	0.4	0.3	0.5	0.4	0.5	0.6	0.5	1.6	1.9	1.3	1.3	1.6	1.5	1.6	1.5	2.2	1.7
Equivalised income																	
Top quintile (%)	19.3	19.4	19.5	19.3	20.4	19.7	20.4	18.7	17.9	16.6	18.8	20.7	20.5	17.9	18.5	17.5	16.7
4th (%)	19.7	19.8	17.5	18.5	17.8	17.9	19.0	17.8	16.8	19.4	17.8	19.4	18.0	17.9	17.1	19	18.3
3rd (%)	17.8	16.9	16.4	16.1	14.8	15.1	14.4	16.4	15.5	15.8	14.3	16.4	16.3	16	13.7	14.3	15.8
2nd (%)	13.5	12.6	13.9	13.6	13.8	14.9	13.6	14.3	15.2	12.7	13.5	11.0	13.0	12.8	14.7	14.2	14.9
Bottom quintile (%)	14.4	15.9	14.4	12.1	11.5	12.4	13	12.3	12.8	14.7	14.8	13.8	12.8	14.7	14.2	16.3	13.7
Unknown (%)	15.3	15.4	18.3	20.4	21.7	20	19.5	20.7	21.9	20.8	20.8	18.7	19.4	20.7	21.8	18.6	20.6
Women Total, n	8234	3825	5674	7818	3812	8339	2537	4718	4788	4610	4870	4489	4456	4459	4461	4509	4530
Age group																	
16–24 (%)	13.5	13.7	13.8	13.9	13.8	14.2	14.3	14.1	14.1	14.1	14.0	13.7	13.3	13	12.8	12.7	12.7
25–34 (%)	16.8	16.4	16.1	15.9	16.0	15.8	15.6	15.8	15.8	16.5	16.5	16.6	16.6	16.6	16.5	16.5	16.3
35–44 (%)	18.9	19.0	18.8	18.9	18.9	18.7	18.4	17.6	17.6	17.1	16.7	16.3	15.9	15.8	15.8	15.7	15.7
45–54 (%)	15.7	15.7	15.6	15.6	15.9	16.0	16.3	16.9	16.9	17.0	17.1	17.3	17.4	17.4	17.3	17.2	16.8
55–64 (%)	14.1	14.3	14.3	14.4	14.7	14.6	14.6	14.6	14.6	14.4	14.0	13.9	14.0	14.2	14.5	14.8	15.1
65–74 (%)	10.7	10.7	10.5	10.5	10.5	10.4	10.5	10.9	10.9	10.9	11.3	11.7	12.2	12.4	12.5	12.5	12.5
75 + (%)	10.3	10.2	10.9	10.9	10.3	10.2	10.2	10.2	10.2	10.0	10.4	10.4	10.5	10.5	10.5	10.6	10.8
Ethnicity																	
White (%)	91.3	91.0	90.8	89.5	87.7	89.0	89.2	88.6	87.8	87.4	86.8	86.4	87.3	86.3	85.3	84.6	83.5
Black (%)	2.4	2.6	2.2	2.6	3.2	2.8	3.0	3.1	2.7	3.2	2.7	2.8	3.2	3.5	3.5	4.0	3.4
Asian (%)	4.4	4.5	4.7	5.5	5.8	5.5	5.2	5.3	7.1	7.0	7.7	7.9	6.7	7.4	8.3	8.1	10.1
Mixed (%)	0.7	0.8	1.0	1.0	1.7	1.2	1.1	1.3	1.3	1.4	1.5	1.6	1.5	1.6	1.7	2.1	1.8
Others (%)	1.1	1.0	0.8	1.2	1.1	1.1	1.1	1.6	0.5	0.7	1.1	1.0	1.0	1.1	0.8	1.0	1.0
Missing/Unknown (%)	0.2	0.0	0.5	0.2	0.4	0.4	0.4	0.2	0.5	0.3	0.3	0.3	0.2	0.2	0.4	0.2	0.3
IMD Deprivation																	
1—Least deprived (%)	22.1	21.9	19.7	19.4	20.9	21.4	19.6	22.2	20	21.1	19.8	21.4	20.7	20.1	20.2	18.3	20.0
2 (%)	19.4	21.5	21.4	21.6	20.5	19.8	20.5	19.4	21.7	20.9	20.7	19.7	19.9	19.5	20.9	19.6	18.7
3 (%)	19.7	17.8	18.9	21.5	21.3	19.9	21.6	19.5	21.7	20.6	21.2	18.9	20.3	21.4	20.0	21.2	20.0
4 (%)	21.3	21.3	21.9	19.8	19.0	19.9	20.6	20.1	18.3	19.7	20.2	20.6	18.8	18.1	19.8	22.2	20.7
5—Most deprived (%)	17.5	17.5	18.2	17.7	18.4	19.0	17.7	18.8	18.3	17.8	18.1	19.5	20.3	21.0	19.2	18.8	20.6
Education																	
Degree or equivalent (%)	15.0	15.6	16.6	18.2	18.0	18.8	19.8	21.0	23.3	25.2	24.7	25.8	26.7	28.1	29.2	28.4	29.8
Below degree (%)	56.4	53.7	52.2	53.4	52	54.6	53.5	55.8	53.1	52.4	53.4	51.5	53.2	50.8	50.7	50.9	50.7
No qualification (%)	28.3	30.4	30.5	28.1	29.4	26.2	26.4	23	23.3	22.1	21.4	22.3	19.7	20.9	19.7	20.2	19.1
Unknown (%)	0.3	0.3	0.6	0.3	0.5	0.4	0.3	0.2	0.4	0.2	0.4	0.4	0.3	0.3	0.4	0.5	0.4
Occupational status																	
Managerial (%)	27.6	28.9	27.8	28.0	27.7	28.6	27.9	30.0	29.7	28.9	30.3	29.6	31.8	30.0	30.9	31.3	33.5
Intermediate (%)	23.5	23.3	23.3	23.6	23.8	23.5	25.3	22.7	25.1	26.8	25.1	25.6	25.6	26.0	26.1	26.3	25
Manual (%)	42.5	41.3	40.5	40.1	39.3	39.8	38.5	38.9	35.0	36.0	35.0	35.2	34.1	34.9	34.9	33.7	32.9
Other (%)	6.1	6.3	7.9	8.2	8.6	7.7	8.1	6.3	7.3	6.0	7.1	7.3	6.1	6.6	5.5	6.5	6.1
Unknown (%)	0.3	0.3	0.4	0.2	0.5	0.3	0.3	2.1	2.9	2.2	2.5	2.3	2.4	2.5	2.6	2.2	2.5
Equivalised income																	
Top quintile (%)	15.4	15.9	16.0	15.9	16.7	16.2	17.8	16.0	15.3	15.3	15.3	17.7	17.7	14.6	15.8	15.2	13.8
4th (%)	17.9	17.0	14.7	16.5	15.4	15.8	17.4	16.7	16.1	17.0	16.7	16.7	15.9	16.3	15.4	16.2	16.8
3rd (%)	18.7	15.8	16.1	16.4	14.2	15.6	14.7	16.0	14.9	15.4	14.9	16.5	16.6	15.4	14.7	14.6	16.3
2nd (%)	14.9	13.4	14.8	17.1	16.1	16.2	15.7	15.5	16.4	15.0	14.9	12.4	15.8	15.4	15.6	16.0	16.4
Bottom quintile (%)	16.8	20.7	18.8	13.4	14.0	15.0	14.4	14.5	14.9	16.1	15.6	15.2	14.6	16.2	16.7	16.6	15.5
Unknown (%)	16.4	17.2	19.5	20.7	23.6	21.2	20.0	21.3	22.4	21.2	22.6	21.6	19.4	22.1	21.8	21.5	21.3

Table 3 provides a summary results, showing change in relative and absolute inequalities for the four behavioural NCD risk factors and their co-occurrence, by SEPs.

**Table 3:** Summary results showing change in relative and absolute inequalities for four behavioural NCD risk factors and their co-occurrence

Behavioural risk factors	Change relative and absolute inequalities by socioeconomic position indicators	
	Men	Women
Alcohol: drinking more than the UK recommended daily guidelines	Deprivation = RII and SII widened Education = NS Employment = RII widened Income = SII widened	Deprivation = RII widened Education = SII widened Employment = RII widened Income = RII widened
Smoking: current cigarette smoker	Deprivation = NS Education = RII widened Employment = RII widened Income = RII widened	Deprivation = NS Education = NS Employment = NS Income = RII widened
Fruit and vegetables: consuming fewer than the recommended five portions of fruit and vegetables per day	Deprivation = NS Education = RII and SII widened Employment = NS Income = NS	Deprivation = RII and SII narrowed Education = NS Employment = SII narrowed Income = RII and SII narrowed
Physical inactivity: being physically inactive	Deprivation = RII and SII widened Education = RII and SII widened Employment = RII and SII widened Income = RII and SII widened	Deprivation = NS Education = RII and SII widened Employment = RII and SII widened Income = RII widened
Multiple risk factors: having two or more risk factors	Deprivation = NS Education = NS Employment = NS Income = NS	Deprivation = NS Education = NS Employment = NS Income = RII and SII widened

“*RII*” Relative Index of Inequality (measure of relative change in inequality), “*SII*” Slope Index of Inequality (measure of absolute change in inequality), “*NS*” Not significant (No significant change in relative and absolute in inequalities)

### Alcohol

Age-adjusted prevalence of participants drinking more than the current UK recommended daily guidelines decreased from 41% in 2007 to 33% in 2019 for men and from 31 to 27% for women (Table 4). Across both sexes, all socioeconomic groups showed a decrease in prevalence of excessive drinking over the study period, however those in the higher SEPs (i.e. least deprived, degree educated, managerial jobs, or top income) consistently had higher prevalence of excessive alcohol use than those in the lower SEPs. Time trends analysis showed that for men, deprivation inequalities in alcohol consumption increased significantly on both the relative scale (*p* = 0.002) and absolute scale (*p* = 0.041) whilst for women, although there was a widening of inequalities on both relative (*p* = 0.021) and absolute scales (*p* = 0.080), the widening was significant on the relative scale only. Occupational status inequalities increased for both sexes on a relative scale but remained stable on the absolute scale. Income inequalities increased on the absolute scale for men and on a relative scale for women. Education inequalities increased on the absolute scale for women.

**Table 4:** Alcohol: Sex stratified Age-adjusted Prevalence of men and women drinking more than the UK recommended daily guidelines and age-adjusted RII and SII by deprivation, education, occupation and income (prevalence weighted for non-responses & cluster sampling). RII = Relative Index of Inequality (measure of relative change in inequality). SII = Slope Index of Inequality (measure of absolute change in inequality)

Variables	2007	2008	2009	2010	2011	2012	2013	2014	2015	2016	2017	2018	2019	*P* for trend
Men Total	41.1	40	42.3	39.8	37.8	36.4	36.6	36.3	34.4	33.7	33.9	33.4	32.9	
IMD Deprivation														
1-Least deprived	40.5	42.2	44.5	41.5	40.5	38.1	43.0	39.0	35.9	34.4	39.8	38.8	38.7	
2	44.8	45.0	47.0	43.1	44.7	42.7	37.4	40.0	38.4	35.3	36.2	35.6	39.5	
3	39.5	41.3	44.5	40.3	36.3	36.1	38.3	36.2	33.4	37.5	33.4	36.0	32.1	
4	41.8	37.9	38.7	40.2	35.4	33.5	35.7	34.0	31.9	34.8	32.4	28.9	27.9	
5-Most deprived	37.3	34.0	37.0	35.1	32.6	29.7	28.8	33.0	32.7	28.0	28.6	28.6	27.7	
**RII (95% CI)**	0.9 (0.8, 1.1)	0.7 (0.7, 0.8)	0.7 (0.6, 0.9)	0.8 (0.7, 0.9)	0.7 (0.6, 0.9)	0.7 (0.6, 0.8)	0.6 (0.5, 0.8)	0.8 (0.6, 0.9)	0.8 (0.7, 1.0)	0.8 (0.6, 1.0)	0.7 (0.5, 0.8)	0.7 (0.5, 0.8)	0.6 (0.5, 0.7)	0.002
**SII (95% CI)**	0 (-0.1, 0.0)	-0.1 (-0.2, -0.1)	-0.1 (-0.2, 0.0)	-0.1 (-0.2, 0.0)	-0.1 (-0.2, -0.1)	-0.1 (-0.2, -0.1)	-0.2 (-0.2, -0.1)	-0.1 (-0.2, 0.0)	-0.1 (-0.1, 0.0)	-0.1 (-0.2, 0.0)	-0.1 (-0.2, -0.1)	-0.1 (-0.2, -0.1)	-0.2 (-0.2, -0.1)	0.041
Education														
Degree or equivalent	44.7	42.2	42.7	42.0	42.8	38.6	38.5	39.6	34.5	35.1	35.9	37.7	36.4	
Below degree	43.7	41.9	44.6	41.0	38.2	38.6	38.7	38.5	37.1	36.5	35.6	35.0	35.4	
No qualification	33.8	31.0	37.1	32.9	30.5	25.6	25.7	27.4	24.5	25.0	24.2	23.9	21.7	
**RII (95% CI)**	0.8 (0.7, 0.9)	0.8 (0.7, 0.9)	0.8 (0.7, 1.1)	0.8 (0.7, 1.0)	0.8 (0.6, 1.0)	0.7 (0.6, 0.9)	0.8 (0.6, 0.9)	0.8 (0.6, 0.9)	0.8 (0.7, 1.0)	0.8 (0.7, 1.0)	0.7 (0.6, 0.9)	0.7 (0.6, 0.9)	0.7 (0.6, 0.8)	0.563
**SII (95% CI)**	-0.1 (-0.2, 0.0)	-0.1 (-0.2, -0.1)	-0.1 (-0.2, 0.0)	-0.1 (-0.1, 0.0)	-0.1 (-0.2, 0.0)	-0.1 (-0.2, -0.1)	-0.1 (-0.2, 0.0)	-0.1 (-0.2, 0.0)	-0.1 (-0.2, 0.0)	-0.1 (-0.1, 0.0)	-0.1 (-0.2, 0.0)	-0.1 (-0.2, -0.1)	-0.2 (-0.2, -0.1)	0.222
Occupational status														
Managerial	46.2	44.0	46.2	41.6	43.1	40.3	43.2	41.2	38.3	39.2	39.5	39.3	37.8	
Intermediate	43.7	41.2	43.3	42.4	37.9	38.9	36.2	39.0	33.3	34.0	34.9	33.1	34.4	
Routine	39.1	37.4	39.5	38.7	36.0	35.5	33.7	34.1	32.6	31.6	30.6	31.2	30.5	
Other	17.8	18.8	34.0	32.5	37.0	28.6	19.9	21.1	9.6	12.5	29.6	12.6	15.2	
**RII (95% CI)**	0.8 (0.7, 1.0)	0.8 (0.7, 0.9)	0.7 (0.6, 0.9)	0.8 (0.7, 1.0)	0.7 (0.6, 0.9)	0.8 (0.7, 1.0)	0.6 (0.5, 0.8)	0.7 (0.6, 0.9)	0.8 (0.6, 0.9)	0.7 (0.6, 0.9)	0.7 (0.6, 0.8)	0.7 (0.5, 0.8)	0.7 (0.6, 0.9)	0.02
**SII (95% CI)**	-0.1 (-0.2, 0.0)	-0.1 (-0.2, -0.1)	-0.1 (-0.2, 0.0)	-0.1 (-0.2, 0.0)	-0.1 (-0.2, -0.1)	-0.1 (-0.2, 0.0)	-0.2 (-0.2, -0.1)	-0.1 (-0.2, -0.1)	-0.1 (-0.2, 0.0)	-0.1 (-0.2, -0.1)	-0.1 (-0.2, -0.1)	-0.1 (-0.2, -0.1)	-0.1 (-0.2, -0.1)	0.284
Equivalised income														
Top quintile	54.5	49.6	51.3	47.1	52.9	45.6	48.0	44.4	42.8	45.8	40.6	44.8	43.9	
4th	47.9	47.5	42.3	45.2	47.2	42.6	42.3	40.5	37.8	35.6	40.4	36.4	40.1	
3rd	38.6	37.9	44.6	39.0	35.7	35.1	35.1	37.6	32.9	32.4	33.2	33.6	32.3	
2nd	30.3	33.6	37.5	36.7	32.0	29.9	34.3	32.3	30.7	28.6	31.2	28.7	26.8	
Bottom quintile	29.1	29.3	32.6	32.8	30.4	28.6	27.5	25.3	29.5	25.1	25	27.5	22.9	
**RII (95% CI)**	0.4 (0.4, 0.5)	0.5 (0.5, 0.6)	0.6 (0.5, 0.7)	0.6 (0.5, 0.7)	0.5 (0.4, 0.6)	0.5 (0.4, 0.6)	0.5 (0.4, 0.6)	0.5 (0.5, 0.7)	0.6 (0.5, 0.8)	0.5 (0.4, 0.6)	0.5 (0.4, 0.7)	0.5 (0.4, 0.7)	0.5 (0.4, 0.6)	0.833
**SII (95% CI)**	-0.4 (-0.4, -0.3)	-0.3 (-0.3, -0.2)	-0.2 (-0.3, -0.1)	-0.2 (-0.3, -0.1)	-0.3 (-0.4, -0.2)	-0.2 (-0.3, -0.2)	-0.3 (-0.3, -0.2)	-0.2 (-0.3, -0.2)	-0.2 (-0.2, -0.1)	-0.2 (-0.3, -0.2)	-0.2 (-0.3, -0.1)	-0.2 (-0.3, -0.1)	-0.3 (-0.3, -0.2)	0.007
Women Total	31	31.2	30.3	28.1	27.6	28	27.2	25.5	27	27.5	25.2	25.1	27.1	
IMD Deprivation														
1-Least deprived	35.5	35.1	34.1	28.3	29.6	32.6	32.7	30.7	31.2	30.7	28.8	30.3	33.9	
2	34.7	37.0	35.5	33.7	30.2	32.5	28.5	28.2	30.1	32.4	26.2	27.6	33.1	
3	30.4	30.9	31.9	28.5	29.0	28.0	28.6	24.3	28.0	28.4	29.0	25.1	25.1	
4	26.6	27.7	28.5	27.4	26.1	22.9	24.0	23.0	23.6	27.0	22.5	23.2	23.4	
5-Most deprived	25.6	26.6	23.0	22.3	21.3	21.3	21.3	21.5	22.0	20.3	18.1	19.9	21.0	
**RII (95% CI)**	0.7 (0.5, 0.8)	0.6 (0.6, 0.7)	0.6 (0.5, 0.8)	0.7 (0.6, 0.9)	0.7 (0.6, 0.8)	0.6 (0.5, 0.7)	0.6 (0.5, 0.8)	0.6 (0.5, 0.8)	0.6 (0.5, 0.8)	0.6 (0.5, 0.8)	0.6 (0.5, 0.8)	0.6 (0.5, 0.8)	0.5 (0.4, 0.6)	0.021
**SII (95% CI)**	-0.1 (-0.2, -0.1)	-0.1 (-0.2, -0.1)	-0.1 (-0.2, -0.1)	-0.1 (-0.1, 0.0)	-0.1 (-0.2, -0.1)	-0.2 (-0.2, -0.1)	-0.1 (-0.2, -0.1)	-0.1 (-0.2, -0.1)	-0.1 (-0.2, -0.1)	-0.1 (-0.2, -0.1)	-0.1 (-0.2, -0.1)	-0.1 (-0.2, -0.1)	-0.2 (-0.2, -0.1)	0.08
Education														
Degree or equivalent	41.7	35.2	32.1	33.2	33.4	33.5	30.6	32.1	30.5	33.2	28.9	31.7	32.8	
Below degree	33	33.6	32.6	29	28.7	28.8	29.5	26.2	28.4	27.4	26.7	25.9	28.5	
No qualification	20.6	20.7	19.7	17.6	17.2	19.7	15.7	16.5	14.9	17.9	14.1	12.6	15.5	
**RII (95% CI)**	0.5 (0.4, 0.6)	0.6 (0.6, 0.7)	0.7 (0.5, 0.9)	0.6 (0.5, 0.7)	0.6 (0.5, 0.7)	0.6 (0.5, 0.7)	0.7 (0.5, 0.8)	0.5 (0.4, 0.6)	0.6 (0.5, 0.7)	0.5 (0.4, 0.7)	0.7 (0.5, 0.8)	0.5 (0.4, 0.6)	0.6 (0.5, 0.8)	0.476
**SII (95% CI)**	-0.2 (-0.3, -0.2)	-0.2 (-0.2, -0.1)	-0.1 (-0.2, -0.1)	-0.2 (-0.2, -0.1)	-0.2 (-0.2, -0.1)	-0.2 (-0.2, -0.1)	-0.1 (-0.2, -0.1)	-0.2 (-0.2, -0.1)	-0.2 (-0.2, -0.1)	-0.2 (-0.2, -0.1)	-0.1 (-0.2, -0.1)	-0.2 (-0.3, -0.2)	-0.2 (-0.2, -0.1)	*p* < 0.001
Occupational status														
Managerial	37.8	38.5	35.6	33.9	33.8	33.1	32.3	33.8	31.6	35.6	32.0	34.1	35.9	
Intermediate	34.8	32.4	32.0	28.7	29.1	29.9	31.7	27.0	30.8	31.0	26.7	26.8	30.3	
Routine	28.2	28.0	29.3	25.3	24.2	25.3	23.8	21.2	24.3	22.1	21.2	19.7	21.9	
Other	17.4	14.3	10.2	8.5	7.7	14.8	6.6	3.8	7.4	9.7	14.3	5.2	4.5	
**RII (95% CI)**	0.6 (0.5, 0.8)	0.6 (0.5, 0.7)	0.7 (0.6, 0.9)	0.6 (0.5, 0.7)	0.6 (0.5, 0.8)	0.7 (0.5, 0.8)	0.6 (0.5, 0.8)	0.5 (0.4, 0.6)	0.7 (0.6, 0.8)	0.5 (0.4, 0.6)	0.5 (0.4, 0.6)	0.4 (0.3, 0.5)	0.5 (0.4, 0.6)	0.001
**SII (95% CI)**	-0.2 (-0.2, -0.1)	-0.2 (-0.2, -0.1)	-0.1 (-0.2, 0.0)	-0.2 (-0.2, -0.1)	-0.2 (-0.2, -0.1)	-0.1 (-0.2, -0.1)	-0.1 (-0.2, -0.1)	-0.2 (-0.3, -0.2)	-0.1 (-0.2, -0.1)	-0.2 (-0.3, -0.2)	-0.2 (-0.2, -0.1)	-0.2 (-0.3, -0.2)	-0.2 (-0.3, -0.2)	0.13
Equivalised income														
Top quintile	41.2	39.7	38.0	34.2	37.6	38.1	39.1	36.6	35.2	38.0	33.6	35.3	40.0	
4th	36.4	38.4	33.0	30.4	33.1	34.9	32.0	32.0	33.5	30.8	31.2	29.5	31.7	
3rd	33.4	29.9	30.6	30.1	27.8	28.9	28.2	24.1	27.6	27.7	26.2	27.8	29.5	
2nd	27.0	29.7	24.7	24.4	24.6	23.5	19.9	22.2	25.2	22.2	23.3	20.8	24.1	
Bottom quintile	20.0	24.8	26.2	23.7	21.5	20.9	20.0	16.9	16.4	20.3	17.7	18.3	17.0	
**RII (95% CI)**	0.5 (0.4, 0.6)	0.6 (0.5, 0.7)	0.6 (0.4, 0.8)	0.6 (0.5, 0.8)	0.5 (0.4, 0.7)	0.5 (0.4, 0.6)	0.4 (0.3, 0.5)	0.4 (0.3, 0.5)	0.5 (0.4, 0.6)	0.4 (0.3, 0.5)	0.5 (0.4, 0.6)	0.4 (0.4, 0.5)	0.4 (0.4, 0.5)	0.001
**SII (95% CI)**	-0.2 (-0.3, -0.2)	-0.2 (-0.2, -0.1)	-0.2 (-0.3, -0.1)	-0.1 (-0.2, -0.1)	-0.2 (-0.3, -0.1)	-0.2 (-0.3, -0.2)	-0.3 (-0.3, -0.2)	-0.3 (-0.3, -0.2)	-0.2 (-0.3, -0.2)	-0.2 (-0.3, -0.2)	-0.2 (-0.3, -0.2)	-0.2 (-0.3, -0.2)	-0.2 (-0.3, -0.2)	0.326

### Smoking

Age-adjusted prevalence of current cigarette smoking decreased from 25% in 2003 to 18% in 2019 for men and from 24 to 15% for women (Table 5). Those in the lower SEPs consistently had a higher prevalence of smoking. Absolute inequalities in smoking remained stable for all measures of SEPs for both sexes during the study period. However, there was significant widening of relative inequalities by income (for both men, *p* = 0.007 and women, *p* = 0.014), by education (men, *p* = 0.023) and occupational status (men, *p* = 0.031). There was also widening of relative inequality by deprivation for women, but this did not reach statistical significance at the 5% level (*p* = 0.069).

**Table 5:** Smoking: Sex stratified Age-adjusted Prevalence of men and women that are current cigarette smoker and age-adjusted RII and SII by deprivation, education, occupation and income (prevalence weighted for non-responses & cluster sampling) RII = Relative Index of Inequality (measure of relative change in inequality). SII = Slope Index of Inequality (measure of absolute change in inequality)

Variables	2003	2004	2005	2006	2007	2008	2009	2010	2011	2012	2013	2014	2015	2016	2017	2018	2019	*P* for trend
Men Total	25.4	22.9	26.2	23.3	23.1	22.8	23.2	21.3	22.4	21.5	23.4	20.7	18.8	19.7	18.9	18.2	18.3	
IMD Deprivation																		
1-Least deprived	18.1	15.4	17.3	16.0	15.9	15.7	13.4	12.6	13.7	12.4	14.7	14.6	10.1	11.4	12.8	11.4	13.1	
2	20.3	18.2	21.4	19.6	18.6	18.2	18.0	19.0	20.1	19.0	16.8	18.2	12.1	15.1	15.7	13.5	16.4	
3	26.3	23.6	24.4	22.2	24.9	19.4	19.1	21.9	17.6	19.2	23.8	20.5	21.0	16.9	17.1	18.0	16.4	
4	28.2	27.4	32.6	27.6	26.4	27.2	30.7	24.9	25.1	26.2	25.8	23.7	23.5	22.2	22.6	19.3	18.9	
5-Most deprived	36.3	33.4	40.0	33.3	32.5	35.3	37.3	29.2	36.9	32.5	38.3	28.0	28.6	32.1	28.3	30.2	27.1	
**RII (95% CI)**	2.3 (2.0, 2.8)	2.6 (1.9, 3.5)	2.7 (2.1, 3.4)	2.3 (1.9, 2.8)	2.4 (1.8, 3.1)	2.7 (2.2, 3.4)	3.5 (2.5, 5.0)	2.4 (1.8, 3.2)	3.4 (2.5, 4.6)	2.8 (2.1, 3.7)	3 (2.3, 4.1)	2 (1.5, 2.7)	3.4 (2.5, 4.6)	3.7 (2.6, 5.3)	2.6 (1.9, 3.7)	3.1 (2.2, 4.4)	2.3 (1.6, 3.5)	0.526
**SII (95% CI)**	0.2 (0.2, 0.3)	0.2 (0.2, 0.3)	0.3 (0.2, 0.3)	0.2 (0.2, 0.2)	0.2 (0.1, 0.3)	0.2 (0.2, 0.3)	0.3 (0.2, 0.4)	0.2 (0.1, 0.3)	0.3 (0.2, 0.3)	0.2 (0.2, 0.3)	0.3 (0.2, 0.3)	0.2 (0.1, 0.2)	0.2 (0.2, 0.3)	0.2 (0.2, 0.3)	0.2 (0.1, 0.2)	0.2 (0.2, 0.3)	0.2 (0.1, 0.2)	0.38
Education																		
Degree or equivalent	13.2	14.5	14.1	9.8	14.6	11.0	12.9	12.6	10.3	10.6	12.6	11.1	11.5	12.4	11.0	9.7	11.1	
Below degree	25.7	21.6	27.1	24.5	22.2	23.9	22.1	22.6	24.1	22.1	24.5	21.2	20.3	19.7	20.5	20.2	19.1	
No qualification	37.8	35.0	37.6	35.6	36.8	34.6	42.7	31.4	37.9	39.0	36.4	35.4	29.0	36.5	30.7	30.5	30.7	
**RII (95% CI)**	3.1 (2.6, 3.6)	3.1 (2.4, 4.1)	2.9 (2.4, 3.7)	3.6 (3.0, 4.3)	3.7 (2.8, 4.9)	3.5 (3.0, 4.1)	4.9 (3.4, 7.1)	3 (2.3, 4.0)	4.2 (3.4, 5.3)	5.1 (3.9, 6.6)	3.4 (2.7, 4.4)	4.2 (3.2, 5.5)	3.6 (2.6, 4.8)	5 (3.6, 7.0)	3.8 (2.7, 5.2)	4.3 (3.2, 5.7)	3.8 (2.7, 5.5)	0.023
**SII (95% CI)**	0.3 (0.3, 0.4)	0.3 (0.2, 0.3)	0.3 (0.2, 0.4)	0.3 (0.3, 0.4)	0.3 (0.3, 0.4)	0.3 (0.3, 0.4)	0.4 (0.3, 0.5)	0.3 (0.2, 0.3)	0.4 (0.3, 0.4)	0.4 (0.3, 0.4)	0.3 (0.3, 0.4)	0.3 (0.3, 0.4)	0.2 (0.2, 0.3)	0.3 (0.2, 0.4)	0.3 (0.2, 0.3)	0.3 (0.2, 0.3)	0.2 (0.2, 0.3)	0.441
Occupational status																		
Managerial	17.6	15.8	18.2	16.1	14.4	14.3	14.8	13.5	14.0	12.4	15.7	14.1	13.2	12.6	12.8	11.4	11.3	
Intermediate	26.1	23.4	27.9	21.5	22.2	24.9	24.3	23.2	23.3	21.2	20.8	23.7	21.0	19.8	19.8	21.3	18.2	
Routine	32.3	31.0	35.1	33.2	32.2	31.0	31.6	29.6	31.9	31.1	33.4	27.9	26.5	27.6	26.4	26.5	27.6	
Other	27.2	16.8	16.5	21.7	29.8	28.9	23.1	24.3	18.8	22.4	37.7	27.8	32.2	13.4	16.5	7.6	11.9	
**RII (95% CI)**	2.6 (2.2, 3.1)	2.9 (2.2, 3.9)	3.2 (2.5, 4.0)	3.9 (3.2, 4.9)	3.8 (2.9, 5.1)	3.3 (2.8, 4.0)	3.6 (2.5, 5.2)	3.3 (2.5, 4.4)	3.8 (2.9, 5.1)	4.2 (3.2, 5.5)	3.7 (2.8, 5.0)	3.1 (2.3, 4.1)	3.7 (2.7, 5.1)	3.7 (2.7, 5.1)	3.7 (2.6, 5.1)	4 (3.0, 5.4)	4.8 (3.3, 6.9)	0.031
**SII (95% CI)**	0.3 (0.2, 0.3)	0.3 (0.2, 0.3)	0.3 (0.2, 0.4)	0.3 (0.3, 0.4)	0.3 (0.3, 0.4)	0.3 (0.3, 0.3)	0.3 (0.2, 0.4)	0.3 (0.2, 0.3)	0.3 (0.3, 0.4)	0.3 (0.3, 0.4)	0.3 (0.3, 0.4)	0.3 (0.2, 0.3)	0.3 (0.2, 0.3)	0.3 (0.2, 0.3)	0.3 (0.2, 0.3)	0.3 (0.2, 0.3)	0.3 (0.2, 0.3)	0.933
Equivalised income																		
Top quintile	18.5	18.4	17.3	15.4	14.7	16.2	13.4	14	14.2	15.3	16.6	11.5	11.1	9.6	10.6	11	12.3	
4th	20.5	19.5	22.3	19.4	20.8	17.8	19.5	15.2	17.3	14.1	17.2	17	11.9	17.6	15.9	14.3	14.2	
3rd	26.1	22.6	26.6	25.1	22.1	20.2	22.6	20.3	22.4	17.7	17.2	21.8	16.9	22.3	17.7	18.2	16.6	
2nd	29.6	26.5	33.2	28.4	30.0	29	32.3	28.3	27.7	26.6	34.3	30.1	28.9	25.8	27.0	22.5	24.5	
Bottom quintile	37.4	34.1	39.2	34.9	38.3	38.3	37.8	32.3	37.2	34.1	39.0	35.6	32.8	29.1	31.2	26.7	30.2	
**RII (95% CI)**	2.5 (2.1, 2.9)	2.4 (1.8, 3.2)	2.5 (2.0, 3.2)	2.7 (2.3, 3.3)	3.3 (2.5, 4.5)	3.1 (2.5, 3.8)	3.4 (2.3, 5.0)	3.1 (2.3, 4.2)	3.5 (2.7, 4.7)	3.9 (2.8, 5.5)	3.6 (2.7, 4.8)	3.8 (2.8, 5.2)	4.9 (3.5, 6.8)	2.9 (2.2, 3.8)	3.4 (2.4, 4.7)	2.9 (2.2, 3.9)	3.3 (2.3, 4.7)	0.007
**SII (95% CI)**	0.2 (0.2, 0.3)	0.2 (0.2, 0.3)	0.3 (0.2, 0.4)	0.3 (0.2, 0.3)	0.3 (0.2, 0.4)	0.3 (0.2, 0.3)	0.3 (0.2, 0.4)	0.3 (0.2, 0.3)	0.3 (0.2, 0.4)	0.3 (0.2, 0.3)	0.3 (0.2, 0.4)	0.3 (0.2, 0.4)	0.3 (0.2, 0.4)	0.2 (0.2, 0.3)	0.3 (0.2, 0.3)	0.2 (0.2, 0.3)	0.2 (0.2, 0.3)	0.212
Women Total	24	22.5	23.4	21.1	20.7	19.7	20.2	18.2	18.5	17.5	17.3	16.6	16.8	15.5	15.6	15.3	14.8	
IMD Deprivation																		
1-Least deprived	16.6	18.2	16.3	12.9	12.9	12.6	12.9	10.3	11.4	9.1	9.2	9.0	10.9	7.7	8.4	8.7	7.9	
2	17.7	16.4	20.7	17.5	13.9	15.2	17.0	14.9	14.5	16.1	13.4	12.4	12.8	11.4	12.7	11.8	11.8	
3	24.1	20.1	20.7	20.2	20.6	18.2	21.4	19.9	17.3	14.5	17.1	15.7	14.4	13.7	14	14.7	12.6	
4	30.0	26.3	26.1	25.4	24.9	24.6	23.3	22.2	20.4	22.2	20.3	21.0	19.3	21.8	19.0	16.4	17.5	
5-Most deprived	34.3	34.1	34.8	31.2	32.7	29.6	28.2	25.9	32.1	28.6	28.3	25.6	27.0	22.9	24.6	26.2	23.1	
**RII (95% CI)**	2.8 (2.3, 3.3)	2.7 (1.9, 3.7)	2.5 (2.0, 3.2)	2.8 (2.3, 3.4)	3.3 (2.5, 4.4)	2.9 (2.4, 3.5)	2.5 (1.9, 3.4)	2.6 (2.0, 3.3)	3.8 (2.9, 5.0)	3.6 (2.7, 4.8)	3.5 (2.6, 4.7)	3.5 (2.7, 4.7)	3.4 (2.4, 5.0)	3.6 (2.7, 4.8)	3.4 (2.5, 4.6)	3.8 (2.7, 5.5)	3.5 (2.6, 4.8)	0.069
**SII (95% CI)**	0.2 (0.2, 0.3)	0.2 (0.1, 0.3)	0.2 (0.2, 0.3)	0.2 (0.2, 0.3)	0.2 (0.2, 0.3)	0.2 (0.2, 0.2)	0.2 (0.1, 0.2)	0.2 (0.1, 0.2)	0.2 (0.2, 0.3)	0.2 (0.2, 0.3)	0.2 (0.2, 0.3)	0.2 (0.2, 0.3)	0.2 (0.1, 0.2)	0.2 (0.2, 0.2)	0.2 (0.1, 0.2)	0.2 (0.2, 0.2)	0.2 (0.1, 0.2)	0.435
Education																		
Degree or equivalent	13.4	10.4	14.1	10.4	11.6	10.8	8.4	7.2	7.7	8.0	9.4	9.8	8.4	8.2	6.9	8.0	8.3	
Below degree	23.1	21.3	23.3	21.0	19.5	19.7	20.4	19.2	20.3	19.3	18.6	17.8	17.9	17.6	19.0	17.2	16.6	
No qualification	33.2	34.9	29.1	30.6	31.3	30.5	30.0	30.2	27.1	26.6	27.7	27.1	29.4	25.0	23.0	23.0	26.6	
**RII (95% CI)**	2.8 (2.4, 3.4)	4 (3.1, 5.1)	2.4 (2.0, 3.0)	3.4 (2.8, 4.1)	3.7 (2.9, 4.8)	3.8 (3.2, 4.5)	3.6 (2.7, 4.8)	4.6 (3.4, 6.2)	3.8 (3.0, 4.9)	4.1 (3.2, 5.2)	3.7 (2.9, 4.9)	4 (3.0, 5.2)	4.5 (3.3, 6.0)	4.1 (3.1, 5.6)	4.6 (3.4, 6.1)	3.7 (2.8, 5.0)	4.5 (3.2, 6.3)	0.141
**SII (95% CI)**	0.3 (0.2, 0.3)	0.3 (0.3, 0.4)	0.2 (0.2, 0.3)	0.3 (0.2, 0.3)	0.3 (0.2, 0.4)	0.3 (0.2, 0.3)	0.3 (0.2, 0.4)	0.3 (0.3, 0.4)	0.3 (0.2, 0.3)	0.3 (0.2, 0.3)	0.3 (0.2, 0.3)	0.3 (0.2, 0.3)	0.3 (0.2, 0.3)	0.2 (0.2, 0.3)	0.3 (0.2, 0.3)	0.2 (0.2, 0.3)	0.2 (0.2, 0.3)	0.401
Occupational status																		
Managerial	19.3	14.3	20.3	15.5	14.0	15.5	14.2	11.0	13.9	10.6	13.5	12.9	11.4	9.3	9.7	9.4	10.8	
Intermediate	21.0	20.4	20.8	18.8	18.4	17.8	19.1	18.6	15.3	16.8	15.1	13.4	15.6	15.1	13.0	14.5	12.0	
Routine	30.8	31.2	28.7	28.7	28.5	25.9	26.9	24.6	28.0	26.8	24.9	24.4	24.7	23.8	24.1	23.7	23.5	
Other	17.9	19.7	17.4	15.5	18.7	18.4	20.4	15.1	9.7	7.6	9.3	9.9	7.3	5.8	4.1	6.2	3.9	
**RII (95% CI)**	2.5 (2.0, 3.1)	4.1 (3.0, 5.7)	2 (1.5, 2.6)	3.3 (2.6, 4.1)	3.9 (2.8, 5.4)	2.8 (2.3, 3.4)	2.9 (2.0, 4.4)	3.5 (2.7, 4.7)	4.6 (3.3, 6.6)	5.2 (3.7, 7.2)	3.4 (2.4, 4.7)	4 (2.8, 5.5)	3.8 (2.8, 5.3)	5 (3.5, 7.1)	6 (4.1, 8.7)	5.4 (3.7, 7.7)	4.8 (3.1, 7.3)	0.109
**SII (95% CI)**	0.2 (0.2, 0.3)	0.3 (0.2, 0.4)	0.2 (0.1, 0.2)	0.2 (0.2, 0.3)	0.3 (0.2, 0.3)	0.2 (0.2, 0.2)	0.2 (0.2, 0.3)	0.2 (0.2, 0.3)	0.3 (0.2, 0.3)	0.3 (0.2, 0.3)	0.2 (0.1, 0.3)	0.2 (0.2, 0.3)	0.2 (0.2, 0.3)	0.2 (0.2, 0.3)	0.3 (0.2, 0.3)	0.2 (0.2, 0.3)	0.2 (0.2, 0.3)	0.111
Equivalised income																		
Top quintile	17.1	15.9	16.1	13.1	12.7	11.3	11.5	9.3	9.2	9.4	10.5	9.6	8.1	7.1	8.2	9.1	7.9	
4th	19.7	18.1	18.8	18.1	12.1	14.9	15.7	14.5	13.9	10.6	14.1	12.7	13.2	10.6	8.7	10.8	8.5	
3rd	24.3	21.7	24.4	20.3	21.6	19.5	21.7	17.4	19.4	17.6	14.4	17.4	18.4	15.9	16.5	15.8	15.2	
2nd	29.4	26.9	27.8	27.7	29.1	26.1	22.1	23.3	24.8	22.6	22.1	21.8	22.2	21.2	22.6	19.1	20.3	
Bottom quintile	34.2	34.4	36.7	30.0	31.2	35.2	33.1	29.3	28.3	28.5	30.5	25.0	25.1	24.6	24.8	23.7	23.7	
**RII (95% CI)**	2.5 (2.1, 3.0)	3.1 (2.4, 4.0)	3 (2.4, 3.8)	2.8 (2.3, 3.4)	4 (3.1, 5.1)	4.6 (3.8, 5.6)	3.9 (2.7, 5.7)	3.7 (2.9, 4.8)	3.5 (2.6, 4.6)	4.3 (3.2, 5.8)	4.1 (3.1, 5.5)	3.5 (2.7, 4.7)	3.3 (2.5, 4.4)	4.1 (3.0, 5.5)	4.1 (3.0, 5.5)	3.5 (2.6, 4.8)	4.3 (3.1, 6.0)	0.014
**SII (95% CI)**	0.2 (0.2, 0.3)	0.3 (0.2, 0.3)	0.3 (0.2, 0.3)	0.2 (0.2, 0.3)	0.3 (0.3, 0.4)	0.3 (0.3, 0.4)	0.3 (0.2, 0.3)	0.3 (0.2, 0.3)	0.3 (0.2, 0.3)	0.3 (0.2, 0.3)	0.2 (0.2, 0.3)	0.2 (0.2, 0.3)	0.2 (0.2, 0.3)	0.2 (0.2, 0.3)	0.2 (0.2, 0.3)	0.2 (0.2, 0.3)	0.2 (0.2, 0.3)	0.809

### Fruit and vegetables

In 2003, age-adjusted prevalence of participants consuming fewer than the recommended five portions of fruit and vegetables daily was 78% for men and 74% for women. These improved slightly over the course of the study, but remained common at 75% for men and 70% for women in 2018 (Table 6). Those in the lower SEPs were consistently more likely to have low fruit and vegetable consumption.

**Table 6:** Fruit & Veg: Sex stratified Age-adjusted Prevalence of men and women consuming fewer than the recommended five portions of fruit and vegetables per day and age-adjusted RII and SII by deprivation, education, occupation and income (prevalence weighted for non-responses & cluster sampling). RII = Relative Index of Inequality (measure of relative change in inequality). SII = Slope Index of Inequality (measure of absolute change in inequality)

Variables	2003	2004	2005	2006	2007	2008	2009	2010	2011	2013	2015	2016	2017	2018	*P* for trend
Men Total	77.7	76.5	73.6	72	72.1	74.5	75	74.4	75.1	74.8	75.4	75.6	73.7	75.1	
IMD Deprivation															
1-Least deprived	73.8	71.7	67.4	69.1	68.0	71.8	74.4	72.1	73.2	71.7	72.7	75.6	73.2	73.7	
2	75.3	76.7	73.7	70.6	70.3	72.8	69.8	72.6	71.4	72.2	75.5	73.8	73.5	73.2	
3	78.9	74.7	73.5	71.6	72.7	73.9	75.1	76.3	75.5	75.8	74.4	73.2	74.0	76.5	
4	78.6	78.7	76.4	73.9	73.4	76.3	76.5	73.1	76.7	76.4	77.5	76.4	72.8	74.9	
5-Most deprived	84.0	81.4	79.4	77.2	77.7	78.7	82.4	78.8	80.6	78.4	78.2	80.2	77.0	78.0	
**RII (95% CI)**	1.2 (1.1, 1.2)	1.1 (1.0, 1.2)	1.2 (1.1, 1.3)	1.1 (1.0, 1.2)	1.1 (1.0, 1.2)	1.1 (1.1, 1.2)	1.1 (1.0, 1.3)	1.1 (1.0, 1.2)	1.1 (1.0, 1.2)	1.1 (1.0, 1.2)	1.1 (1.0, 1.2)	1.1 (1.0, 1.2)	1 (0.9, 1.1)	1.1 (1.0, 1.1)	0.153
**SII (95% CI)**	0.1 (0.1, 0.2)	0.1 (0.0, 0.2)	0.1 (0.1, 0.2)	0.1 (0.0, 0.1)	0.1 (0.0, 0.2)	0.1 (0.0, 0.1)	0.1 (0.0, 0.2)	0.1 (0.0, 0.1)	0.1 (0.0, 0.2)	0.1 (0.0, 0.1)	0.1 (0.0, 0.1)	0.1 (0.0, 0.1)	0 (0.0, 0.1)	0 (0.0, 0.1)	0.13
Education															
Degree or equivalent	67.5	66.6	59.4	61.3	57.8	61.1	63.8	59.3	63.7	64.7	64.0	66.3	60.4	65.4	
Below degree	77.1	78.9	75.8	73.3	72.6	76.3	76.6	77.8	76.4	76.9	77.9	78.4	77.0	77.1	
No qualification	86.0	77.0	79.5	78.1	80.6	81.6	83.6	79.4	81.9	81.6	81.0	81.5	81.7	82.7	
**RII (95% CI)**	1.4 (1.3, 1.4)	1.2 (1.1, 1.3)	1.4 (1.3, 1.5)	1.4 (1.3, 1.5)	1.5 (1.4, 1.6)	1.4 (1.3, 1.5)	1.5 (1.3, 1.6)	1.4 (1.3, 1.6)	1.4 (1.3, 1.5)	1.4 (1.3, 1.5)	1.4 (1.3, 1.6)	1.3 (1.2, 1.5)	1.5 (1.3, 1.6)	1.4 (1.3, 1.5)	*p* < 0.001
**SII (95% CI)**	0.2 (0.2, 0.3)	0.1 (0.1, 0.2)	0.2 (0.2, 0.3)	0.2 (0.2, 0.3)	0.3 (0.2, 0.4)	0.3 (0.2, 0.3)	0.3 (0.2, 0.4)	0.3 (0.2, 0.3)	0.3 (0.2, 0.3)	0.2 (0.2, 0.3)	0.3 (0.2, 0.3)	0.2 (0.2, 0.3)	0.3 (0.2, 0.4)	0.3 (0.2, 0.3)	*p* < 0.001
Occupational status															
Managerial	72.2	72.5	66.9	66.0	65.0	67.1	69.2	69.7	69.0	68.9	70.6	71.0	65.0	69.8	
Intermediate	79.0	71.3	73.4	70.7	71.8	77.7	75.0	74.7	75.8	75.7	74.6	74.5	73.4	74.5	
Routine	82.4	82.9	80.3	77.9	78.0	79.9	81.1	79.8	81.0	79.7	80.8	81.2	80.2	81.6	
Other	84.7	65.2	62.3	75.4	73.8	73.7	83.4	72.0	86	83.6	85.9	45.9	96.3	73.3	
**RII (95% CI)**	1.2 (1.2, 1.3)	1.3 (1.2, 1.4)	1.4 (1.3, 1.5)	1.3 (1.2, 1.4)	1.4 (1.3, 1.5)	1.3 (1.3, 1.4)	1.3 (1.2, 1.5)	1.3 (1.2, 1.4)	1.3 (1.2, 1.4)	1.3 (1.2, 1.4)	1.3 (1.2, 1.4)	1.3 (1.2, 1.4)	1.4 (1.3, 1.5)	1.3 (1.2, 1.4)	0.429
**SII (95% CI)**	0.2 (0.1, 0.2)	0.2 (0.1, 0.3)	0.2 (0.2, 0.3)	0.2 (0.2, 0.2)	0.2 (0.2, 0.3)	0.2 (0.2, 0.3)	0.2 (0.1, 0.3)	0.2 (0.1, 0.2)	0.2 (0.2, 0.3)	0.2 (0.1, 0.3)	0.2 (0.1, 0.2)	0.2 (0.1, 0.2)	0.2 (0.2, 0.3)	0.2 (0.1, 0.3)	0.518
Equivalised income															
Top quintile	71.4	68.8	63.7	63.8	65.5	66.5	67.2	66.2	69.1	69.3	66.6	70.9	67.9	68.3	
4th	75.1	74.6	72.7	70.7	64.8	72.3	73.2	71.1	70.7	70.4	75.0	72.8	71.9	75.9	
3rd	77.7	80.3	73.7	74.0	71.7	77.4	75.3	75.4	78.8	77.1	74.4	74.1	76.2	77.2	
2nd	81.7	83.6	78.1	79.6	79.6	79.5	79.6	82.3	81.6	78.7	80.7	79.6	76.8	78.2	
Bottom quintile	84.4	80.1	82.2	78.0	79.6	79.6	82.4	80.1	79.2	81.3	79.6	78.3	80.0	77.7	
**RII (95% CI)**	1.2 (1.2, 1.3)	1.2 (1.1, 1.3)	1.3 (1.2, 1.5)	1.3 (1.2, 1.4)	1.4 (1.2, 1.5)	1.2 (1.1, 1.3)	1.2 (1.1, 1.4)	1.3 (1.2, 1.4)	1.2 (1.1, 1.3)	1.2 (1.1, 1.3)	1.2 (1.1, 1.3)	1.2 (1.1, 1.3)	1.2 (1.1, 1.4)	1.1 (1.0, 1.2)	0.34
**SII (95% CI)**	0.2 (0.1, 0.2)	0.2 (0.1, 0.2)	0.2 (0.2, 0.3)	0.2 (0.1, 0.2)	0.2 (0.2, 0.3)	0.2 (0.1, 0.2)	0.2 (0.1, 0.3)	0.2 (0.1, 0.2)	0.2 (0.1, 0.2)	0.1 (0.1, 0.2)	0.2 (0.1, 0.2)	0.1 (0.1, 0.2)	0.2 (0.1, 0.2)	0.1 (0.0, 0.2)	0.246
Women Total	73.9	73	70.1	68.3	69.2	70.8	72.1	73.1	71.2	72.2	73	71.8	68.4	69.9	
IMD Deprivation															
1-Least deprived	67.7	65.7	63.9	62.6	63.0	68.2	64.4	67.8	70.8	68.2	69.7	69.1	65.3	66.7	
2	68.8	72.0	69.0	66.8	69.8	67.7	71.5	73.0	66.7	72.3	70.3	67.6	66.7	69.6	
3	74.3	72.6	70.5	67.7	69.1	69.3	74.0	72.0	73.0	73.2	70.5	70.4	68.3	67.5	
4	78.2	76.8	70.5	68.2	72.3	71.4	73.3	75.1	72.2	71.4	74.6	74.2	70.2	70.8	
5-Most deprived	83.1	80.0	78.4	78.1	73.1	79.8	79.6	80.1	76.2	76.9	80.7	78.1	73.8	75.0	
**RII (95% CI)**	1.3 (1.2, 1.4)	1.3 (1.2, 1.4)	1.2 (1.1, 1.3)	1.2 (1.2, 1.3)	1.2 (1.1, 1.3)	1.2 (1.1, 1.3)	1.2 (1.1, 1.4)	1.2 (1.1, 1.3)	1.1 (1.1, 1.2)	1.1 (1.0, 1.2)	1.2 (1.1, 1.3)	1.2 (1.1, 1.3)	1.2 (1.1, 1.3)	1.1 (1.0, 1.2)	0.006
**SII (95% CI)**	0.2 (0.2, 0.2)	0.2 (0.1, 0.2)	0.1 (0.1, 0.2)	0.1 (0.1, 0.2)	0.1 (0.0, 0.2)	0.1 (0.1, 0.2)	0.2 (0.1, 0.2)	0.1 (0.1, 0.2)	0.1 (0.0, 0.1)	0.1 (0.0, 0.1)	0.1 (0.1, 0.2)	0.1 (0.1, 0.2)	0.1 (0.1, 0.2)	0.1 (0.0, 0.1)	0.003
Education															
Degree or equivalent	57.6	56.3	55.7	54.4	55.0	56.6	52.9	57.4	60.8	60.0	58.4	60.6	54.4	60.2	
Below degree	72.7	70.8	69.9	67.7	67.7	70.8	72.5	73.4	72.0	74.3	75.5	74.1	71.2	71.8	
No qualification	82.4	82.8	76.7	76.0	77.6	79.7	84.9	79.4	78.8	76.9	80.8	77.8	80	76.1	
**RII (95% CI)**	1.5 (1.4, 1.6)	1.6 (1.4, 1.7)	1.5 (1.3, 1.6)	1.5 (1.4, 1.7)	1.5 (1.4, 1.7)	1.5 (1.5, 1.6)	1.7 (1.6, 1.9)	1.5 (1.4, 1.6)	1.4 (1.3, 1.5)	1.4 (1.3, 1.6)	1.5 (1.4, 1.7)	1.4 (1.3, 1.5)	1.7 (1.6, 1.9)	1.4 (1.3, 1.5)	0.796
**SII (95% CI)**	0.3 (0.3, 0.4)	0.3 (0.3, 0.4)	0.3 (0.2, 0.3)	0.3 (0.3, 0.4)	0.3 (0.2, 0.4)	0.3 (0.3, 0.4)	0.4 (0.3, 0.5)	0.3 (0.2, 0.4)	0.3 (0.2, 0.3)	0.3 (0.2, 0.3)	0.3 (0.3, 0.4)	0.3 (0.2, 0.3)	0.4 (0.3, 0.4)	0.2 (0.2, 0.3)	0.614
Occupational status															
Managerial	64.6	63.5	61.6	60.7	61.5	62.7	64.7	65.9	64.2	66.9	62.5	64.8	61.0	63.8	
Intermediate	72.5	71.4	68.7	66.4	68.2	70.3	72.9	72.3	70.5	72.4	71.6	71.2	66.5	70.7	
Routine	80.2	80.2	77.8	74.9	75.1	77.4	78.6	79.2	77.8	76.7	81.1	76.4	76.7	76.0	
Other	78.4	80.1	68.3	71.3	72.1	71.7	73.9	69.5	72.6	69.5	75.3	78.6	68.2	68.4	
**RII (95% CI)**	1.4 (1.4, 1.5)	1.5 (1.4, 1.6)	1.5 (1.4, 1.7)	1.4 (1.4, 1.6)	1.4 (1.3, 1.5)	1.4 (1.4, 1.5)	1.4 (1.3, 1.6)	1.4 (1.3, 1.5)	1.4 (1.3, 1.5)	1.3 (1.2, 1.4)	1.5 (1.4, 1.6)	1.3 (1.2, 1.4)	1.5 (1.4, 1.7)	1.4 (1.2, 1.5)	0.066
**SII (95% CI)**	0.3 (0.2, 0.3)	0.3 (0.2, 0.4)	0.3 (0.2, 0.4)	0.3 (0.2, 0.3)	0.2 (0.2, 0.3)	0.3 (0.2, 0.3)	0.3 (0.2, 0.4)	0.2 (0.2, 0.3)	0.2 (0.2, 0.3)	0.2 (0.1, 0.2)	0.3 (0.2, 0.4)	0.2 (0.1, 0.2)	0.3 (0.2, 0.3)	0.2 (0.2, 0.3)	0.043
Equivalised income															
Top quintile	58.8	60.2	63.7	61.0	63.9	60.9	63.4	63.3	62.0	65.4	66.9	64.9	58.7	64.1	
4th	68.8	67.2	70.6	63.4	62.9	68.3	73.4	67.1	66.0	71.6	68.5	67.5	68.8	65.2	
3rd	74.3	76.7	69.4	66.9	68.9	71.3	70.2	76.1	71.7	71.5	74.5	69.5	69.3	68.0	
2nd	80.5	79.8	73.2	74.3	76.5	75.3	75.0	79.9	74.4	73.4	76.4	75.7	69.9	75.4	
Bottom quintile	83.0	81.1	78.4	77.4	75.1	78.6	80.4	78.4	79.1	77.4	79.4	78.3	74.7	75.8	
**RII (95% CI)**	1.4 (1.3, 1.5)	1.4 (1.3, 1.5)	1.3 (1.2, 1.4)	1.4 (1.3, 1.5)	1.3 (1.2, 1.4)	1.3 (1.2, 1.4)	1.3 (1.2, 1.4)	1.3 (1.2, 1.4)	1.3 (1.2, 1.4)	1.2 (1.1, 1.3)	1.3 (1.2, 1.4)	1.3 (1.1, 1.4)	1.3 (1.2, 1.4)	1.3 (1.2, 1.4)	0.004
**SII (95% CI)**	0.3 (0.2, 0.3)	0.3 (0.2, 0.3)	0.2 (0.1, 0.2)	0.2 (0.2, 0.3)	0.2 (0.1, 0.3)	0.2 (0.2, 0.2)	0.2 (0.1, 0.3)	0.2 (0.1, 0.3)	0.2 (0.1, 0.3)	0.1 (0.1, 0.2)	0.2 (0.1, 0.2)	0.2 (0.1, 0.2)	0.2 (0.1, 0.2)	0.2 (0.1, 0.2)	0.001

For women, there was narrowing of both relative (*p* = 0.006) and absolute inequalities (*p* = 0.003) by neighbourhood deprivation. Similarly for women, there were narrowing of both relative (*p *= 0.004) and absolute inequalities (*p* = 0.001) by income. Women also saw narrowing of occupational status inequalities on the absolute scale (*p* = 0.043).

Conversely for men, there was widening of both relative (*p* < 0.001) and absolute inequalities (*p* < 0.001) by education. All other measures of SEPs inequalities remained stable during the study period.

### Physical activity

Age-adjusted prevalence of physical inactivity decreased over the study period from 65% in 2003 to 57% in 2016 for men and from 76 to 66% for women (Table 7). In 2003, those in lower SEPs had a lower or similar prevalence of physical inactivity compared with those in the higher SEPs, as indicated by RII of below or near one and SII of below or near zero. However, by the end of the study, all RIIs and SIIs were above one (RII) and zero (SII), indicating that relative and absolute inequalities have widened. The p-values derived from the linear trend test showed that for men, there has been a significant widening of both relative and absolute inequalities for all SEPs. For women, education inequalities and occupational status inequalities has widened on both the relative and absolute scale. Women also saw widening of relative inequality by income (Table 7).

**Table 7:** Physical inactivity: Sex stratified Age-adjusted Prevalence of men and women that are physically inactive and age-adjusted RII and SII by deprivation, education, occupation and income (prevalence weighted for non-responses & cluster sampling). RII = Relative Index of Inequality (measure of relative change in inequality). SII = Slope Index of Inequality (measure of absolute change in inequality)

Variables	2003	2004	2006	2008	2012	2016	*P* for trend
Men Total	65.1	64.3	61.8	59.9	58.5	56.9	
IMD Deprivation							
1-Least deprived	67.5	68.2	63.5	59.5	56.8	53.7	
2	64.9	64.6	59.7	59.1	56.1	57.3	
3	63.4	60.0	59.5	58.8	55.6	55.1	
4	63.3	64.4	60.2	58.7	57.4	56.3	
5-Most deprived	66.8	65.4	67.8	64.5	68.4	62.5	
**RII (95% CI)**	1 (0.9, 1.0)	1 (0.9, 1.1)	1.1 (1.0, 1.1)	1.1 (1.0, 1.2)	1.2 (1.1, 1.3)	1.2 (1.1, 1.3)	0.001
**SII (95% CI)**	0 (-0.1, 0.0)	-0.1 (-0.1, 0.0)	0 (0.0, 0.1)	0 (0.0, 0.1)	0.1 (0.0, 0.2)	0.1 (0.0, 0.2)	*p* < 0.001
Education							
Degree or equivalent	70.6	68.6	61.9	58.5	57.5	54.1	
Below degree	62.9	61.9	59.9	58.3	56.0	54.9	
No qualification	63.2	62.0	63.4	61.7	64.1	65.9	
**RII (95% CI)**	0.9 (0.8, 0.9)	0.8 (0.8, 0.9)	1 (0.9, 1.1)	1.1 (1.0, 1.2)	1.1 (1.0, 1.3)	1.3 (1.2, 1.4)	*p* < 0.001
**SII (95% CI)**	-0.1 (-0.2, -0.1)	-0.1 (-0.2, -0.1)	0 (-0.1, 0.0)	0 (0.0, 0.1)	0.1 (0.0, 0.1)	0.1 (0.1, 0.2)	*p* < 0.001
Occupational status							
Managerial	72.1	70.2	64.7	61.8	58.1	55.8	
Intermediate	61.1	58.9	57.6	57.9	55.4	53.6	
Routine	58.9	59.4	57.9	56.9	56.6	56.1	
Other	82.4	68.6	81.5	72.3	55.2	78.2	
**RII (95% CI)**	0.7 (0.7, 0.8)	0.8 (0.7, 0.8)	0.8 (0.7, 0.9)	0.9 (0.8, 1.0)	0.9 (0.8, 1.0)	1.1 (0.9, 1.2)	*p* < 0.001
**SII (95% CI)**	-0.3 (-0.3, -0.2)	-0.2 (-0.3, -0.1)	-0.2 (-0.2, -0.1)	-0.1 (-0.1, 0.0)	-0.1 (-0.1, 0.0)	0 (-0.1, 0.1)	*p* < 0.001
Equivalised income							
Top quintile	67.0	61.6	59.3	59.2	53.5	50.3	
4th	62.6	61.8	56.2	57.3	54.7	55.3	
3rd	60.5	61.3	58.2	55.0	57.6	56.2	
2nd	63.5	61.4	63.5	59.1	55.5	59.1	
Bottom quintile	71.5	68.4	67.0	68.6	66.2	65.4	
**RII (95% CI)**	1 (1.0, 1.1)	1 (0.9, 1.2)	1.1 (1.0, 1.2)	1.1 (1.1, 1.2)	1.2 (1.1, 1.4)	1.4 (1.2, 1.6)	*p* < 0.001
**SII (95% CI)**	0 (0.0, 0.1)	0 (-0.1, 0.1)	0 (0.0, 0.1)	0.1 (0.0, 0.1)	0.1 (0.0, 0.2)	0.2 (0.1, 0.3)	*p* < 0.001
Women Total	75.8	75.3	71.8	69.7	67.8	65.6	
IMD Deprivation							
1-Least deprived	76.4	72.9	73.0	68.1	64.0	61.1	
2	75.9	74.8	70.6	67.6	64.5	62.4	
3	73.9	73.7	70.6	69.1	68.1	67.1	
4	76.4	77.3	71.2	70.4	69.2	65.7	
5-Most deprived	77.4	78.7	75.1	74.8	74.2	71.5	
**RII (95% CI)**	1 (1.0, 1.1)	1.1 (1.0, 1.2)	1 (1.0, 1.1)	1.1 (1.1, 1.2)	1.2 (1.1, 1.3)	1.2 (1.1, 1.3)	0.065
**SII (95% CI)**	0 (0.0, 0.0)	0.1 (0.0, 0.1)	0 (0.0, 0.1)	0.1 (0.0, 0.1)	0.1 (0.1, 0.2)	0.1 (0.1, 0.2)	0.087
Education							
Degree or equivalent	73.6	71.8	69.1	65.5	60.3	59.9	
Below degree	75.6	74.1	70.9	67.9	67.6	64.9	
No qualification	77.3	77.3	75.3	75.5	74.9	73.4	
**RII (95% CI)**	1.1 (1.0, 1.1)	1.1 (1.0, 1.2)	1.1 (1.0, 1.2)	1.2 (1.1, 1.3)	1.3 (1.2, 1.4)	1.3 (1.2, 1.4)	*p* < 0.001
**SII (95% CI)**	0 (0.0, 0.1)	0.1 (0.0, 0.1)	0.1 (0.0, 0.1)	0.1 (0.1, 0.2)	0.2 (0.1, 0.2)	0.2 (0.1, 0.2)	*p* < 0.001
Occupational status							
Managerial	76.1	71.1	70.3	67.2	62.4	61.7	
Intermediate	77.2	78.1	71.6	69.9	67.8	64.3	
Routine	73.0	74.6	70.2	69.6	69.2	66.0	
Other	84.0	88.5	82.4	79.5	84.2	82.4	
**RII (95% CI)**	0.9 (0.9, 1.0)	1.1 (1.0, 1.2)	1 (0.9, 1.0)	1.1 (1.0, 1.1)	1.2 (1.1, 1.3)	1.1 (1.0, 1.3)	0.011
**SII (95% CI)**	-0.1 (-0.1, 0.0)	0 (0.0, 0.1)	0 (-0.1, 0.0)	0 (0.0, 0.1)	0.1 (0.0, 0.2)	0.1 (0.0, 0.1)	0.047
Equivalised income							
Top quintile	73.5	68.9	71.6	64.9	61.8	56.3	
4th	74.6	72.3	69.3	69.3	67.1	64.7	
3rd	75.3	74.7	69.3	69.8	65.4	63.5	
2nd	74.7	77.9	72.0	71.6	71.9	66.8	
Bottom quintile	77.5	78.4	74.5	72.4	73.0	70.2	
**RII (95% CI)**	1 (1.0, 1.1)	1.2 (1.1, 1.3)	1.1 (1.0, 1.1)	1.1 (1.1, 1.2)	1.2 (1.1, 1.3)	1.2 (1.1, 1.4)	0.046
**SII (95% CI)**	0 (0.0, 0.0)	0.1 (0.0, 0.2)	0 (0.0, 0.1)	0.1 (0.0, 0.1)	0.1 (0.1, 0.2)	0.1 (0.1, 0.2)	0.169

### Multiple risk factors

In 2007, 17% of the study population was estimated to have zero risk factors, 45% had one, 38% had two or three, and 8% had all three risk factors. By 2018, the proportion of the population with one risk factor had increased to 51% and there was improvement in those with two or three (decrease to 31%), all three (decreased to 5%) and zero (increased to 18%) risk factors. Compared with women, men had higher prevalence of two or more risk factors (42% vs 33% in 2007 and 35% vs 26% in 2018) and lower prevalence of zero (15% vs 21% in 2018) or one risk factor (49% vs 53% in 2018).

Overall, after adjusting for age, the proportion of the population with two or more risk factors decreased from 41% in 2007 to 35% in 2018 for men and from 33 to 26% for women (Table 8). For women, there was narrowing of both relative (*p* = 0.009) and absolute inequalities (*p* = 0.025) by income. All other measures of SEPs inequalities remained stable during the study period for both men and women.

**Table 8:** Multiple Risk Factors: Sex stratified Age-adjusted Prevalence of men and women with two or more risk factors and age-adjusted RII and SII by deprivation, education, occupation and income (prevalence weighted for non-responses & cluster sampling). RII = Relative Index of Inequality (measure of relative change in inequality). SII = Slope Index of Inequality (measure of absolute change in inequality)

Variables	2007	2008	2009	2010	2011	2013	2015	2016	2017	2018	*P* for trend
Men Total	40.9	41.3	44.0	41.1	39.7	40.3	37.0	36.4	35.9	35.1	
IMD Deprivation											
1-Least deprived	33.6	37.9	39.5	36.0	36.8	37.8	33.3	32.5	37.3	32.8	
2	39.6	40.8	42.3	42.0	39.9	36.3	34.5	33.0	35.6	32.4	
3	40.3	40.4	40.6	43.3	36.2	41.0	38.6	35.8	34.4	37.0	
4	45.6	42.8	47.9	41.8	40.7	41.8	38.6	38.4	37.3	33.8	
5-Most deprived	47.1	46.3	51.8	43.5	46.1	45.7	42.4	43.3	37.2	40.8	
**RII (95% CI)**	1.4 (1.2, 1.7)	1.2 (1.1, 1.3)	1.3 (1.1, 1.6)	1.1 (1.0, 1.4)	1.3 (1.1, 1.5)	1.2 (1.0, 1.5)	1.3 (1.1, 1.6)	1.4 (1.2, 1.7)	1 (0.8, 1.2)	1.2 (1.0, 1.5)	0.166
**SII (95% CI)**	0.2 (0.1, 0.2)	0.1 (0.0, 0.1)	0.1 (0.0, 0.2)	0.1 (0.0, 0.1)	0.1 (0.0, 0.2)	0.1 (0.0, 0.2)	0.1 (0.0, 0.2)	0.1 (0.1, 0.2)	0 (-0.1, 0.1)	0.1 (0.0, 0.1)	0.05
Education											
Degree or equivalent	32.9	32.1	34.7	34.1	32.0	31.9	28.3	29.3	29.7	30.2	
Below degree	42.6	43.8	45.6	43.6	42.0	43.1	41.3	39.3	39.2	37.5	
No qualification	47.4	45.2	51.6	44.2	45.4	44.6	39.4	44.6	39.7	41.7	
**RII (95% CI)**	1.7 (1.4, 2.0)	1.6 (1.4, 1.8)	1.7 (1.4, 2.1)	1.5 (1.3, 1.8)	1.7 (1.5, 2.0)	1.6 (1.4, 1.9)	1.7 (1.5, 2.0)	1.9 (1.6, 2.4)	1.6 (1.3, 1.9)	1.8 (1.5, 2.2)	0.106
**SII (95% CI)**	0.2 (0.2, 0.3)	0.2 (0.2, 0.3)	0.2 (0.2, 0.3)	0.2 (0.1, 0.2)	0.2 (0.2, 0.3)	0.2 (0.1, 0.3)	0.2 (0.1, 0.3)	0.2 (0.2, 0.3)	0.2 (0.1, 0.2)	0.2 (0.1, 0.3)	0.441
Occupational status											
Managerial	37.6	36.9	40.7	36.9	36.9	37.6	35.4	34.2	33.7	33.6	
Intermediate	40.7	44	42.8	44.5	40.1	38.7	39.3	35.3	36.7	35.1	
Routine	48.3	46.7	50.3	46.6	46.3	46.8	41.9	42.6	41.1	41.3	
Other	33.6	34.5	36.8	35.9	40.4	50.8	31.1	14.5	37.3	12.5	
**RII (95% CI)**	1.7 (1.4, 2.0)	1.5 (1.3, 1.6)	1.4 (1.2, 1.8)	1.4 (1.2, 1.7)	1.6 (1.3, 1.8)	1.5 (1.3, 1.8)	1.4 (1.2, 1.7)	1.5 (1.2, 1.9)	1.5 (1.2, 1.8)	1.5 (1.2, 1.8)	0.28
**SII (95% CI)**	0.2 (0.2, 0.3)	0.2 (0.1, 0.2)	0.2 (0.1, 0.3)	0.1 (0.1, 0.2)	0.2 (0.1, 0.2)	0.2 (0.1, 0.2)	0.1 (0.1, 0.2)	0.2 (0.1, 0.2)	0.1 (0.1, 0.2)	0.1 (0.1, 0.2)	0.061
Equivalised income											
Top quintile	40.0	39.8	43.0	39.4	43.0	41.0	34.7	34.4	34.3	36.5	
4th	40.0	42.5	40.0	40.6	39.1	38.9	35.7	34.8	38.7	34.1	
3rd	39.8	38.2	43.6	39.2	39.6	37.6	34.5	38.6	35.3	36.7	
2nd	41.8	43.6	50.5	44.9	41.0	45.5	42.1	38.7	40.4	35.4	
Bottom quintile	44.4	46.6	48.3	44.3	45.8	46.1	44.1	37.5	37.8	39.3	
**RII (95% CI)**	1.1 (0.9, 1.4)	1.2 (1.0, 1.3)	1.2 (1.0, 1.5)	1.1 (1.0, 1.4)	1.1 (1.0, 1.4)	1.2 (1.0, 1.4)	1.4 (1.2, 1.7)	1.2 (1.0, 1.4)	1.1 (0.9, 1.4)	1.1 (0.9, 1.4)	0.682
**SII (95% CI)**	0.1 (0.0, 0.1)	0.1 (0.0, 0.1)	0.1 (0.0, 0.2)	0.1 (0.0, 0.1)	0 (0.0, 0.1)	0.1 (0.0, 0.1)	0.1 (0.1, 0.2)	0.1 (0.0, 0.1)	0 (0.0, 0.1)	0 (0.0, 0.1)	0.679
Women Total	33.0	32.8	32.8	31.6	30.9	29.7	29.2	28.9	27.4	26.3	
IMD Deprivation											
1-Least deprived	30.3	30.0	29.0	25.5	25.6	26.7	26.8	24.9	23.8	23.8	
2	31.8	31.8	32.1	33.2	29.0	28.9	27.3	29.3	24.7	24.8	
3	31.9	31.8	35.4	33.1	32.7	30.5	27.7	28.1	28.1	25.9	
4	34.8	34.0	33.4	34.0	32.0	29.4	29.2	32.9	28.6	26.6	
5-Most deprived	36.6	38.8	35.6	34.6	37.2	33.6	34.9	30.5	31.1	31.6	
**RII (95% CI)**	1.2 (1.0, 1.4)	1.3 (1.1, 1.4)	1.2 (1.0, 1.5)	1.3 (1.1, 1.5)	1.5 (1.3, 1.7)	1.2 (1.0, 1.5)	1.3 (1.1, 1.6)	1.3 (1.0, 1.6)	1.4 (1.2, 1.7)	1.4 (1.1, 1.7)	0.75
**SII (95% CI)**	0.1 (0.0, 0.1)	0.1 (0.0, 0.1)	0.1 (0.0, 0.1)	0.1 (0.0, 0.1)	0.1 (0.1, 0.2)	0.1 (0.0, 0.1)	0.1 (0.0, 0.1)	0.1 (0.0, 0.1)	0.1 (0.0, 0.1)	0.1 (0.0, 0.1)	0.36
Education											
Degree or equivalent	27.5	25.4	21.4	23.8	26.0	23.2	21.5	24.9	20.9	24.2	
Below degree	33.9	34.5	34.8	33.1	33.0	32.8	31.9	30.6	31.5	28.0	
No qualification	36.2	35.9	36.6	33.8	31.6	30.5	31.0	30.7	28.5	25.3	
**RII (95% CI)**	1.6 (1.3, 1.9)	1.6 (1.4, 1.8)	1.9 (1.5, 2.3)	1.5 (1.3, 1.9)	1.5 (1.2, 1.8)	1.6 (1.4, 2.0)	1.6 (1.4, 1.9)	1.5 (1.2, 1.8)	1.9 (1.5, 2.3)	1.3 (1.1, 1.5)	0.663
**SII (95% CI)**	0.2 (0.1, 0.2)	0.2 (0.1, 0.2)	0.2 (0.2, 0.3)	0.2 (0.1, 0.2)	0.1 (0.1, 0.2)	0.2 (0.1, 0.2)	0.2 (0.1, 0.2)	0.1 (0.0, 0.2)	0.2 (0.1, 0.2)	0.1 (0.0, 0.1)	0.603
Occupational status											
Managerial	30.2	32.5	30.8	28.7	31.3	28.7	25.1	28.2	26.0	26.6	
Intermediate	35.3	33.0	33.8	32.7	30.0	31.4	31.3	30.0	27.0	26.8	
Routine	37.9	36.9	38.8	35.2	36.6	34.3	34.8	32.3	32.5	29.3	
Other	24.2	20.5	17.6	13.1	13.3	13.0	8.5	11.2	13.5	8.7	
**RII (95% CI)**	1.5 (1.2, 1.9)	1.3 (1.1, 1.5)	1.6 (1.3, 2.0)	1.3 (1.1, 1.6)	1.5 (1.3, 1.9)	1.4 (1.2, 1.7)	1.6 (1.4, 2.0)	1.3 (1.1, 1.5)	1.6 (1.2, 2.0)	1.3 (1.0, 1.6)	0.894
**SII (95% CI)**	0.1 (0.1, 0.2)	0.1 (0.0, 0.1)	0.2 (0.1, 0.2)	0.1 (0.0, 0.2)	0.1 (0.1, 0.2)	0.1 (0.1, 0.2)	0.2 (0.1, 0.2)	0.1 (0.0, 0.1)	0.1 (0.1, 0.2)	0.1 (0.0, 0.1)	0.41
Equivalised income											
Top quintile	31.9	30.9	29.4	28.3	29.5	30.4	26.9	27.1	24.4	28.1	
4th	30.4	33.7	33.3	28.8	29.5	30.9	29.3	26.4	27.5	23.7	
3rd	35.1	32.4	32.9	32.6	32.2	29.4	31.9	28.3	30.4	28.4	
2nd	37.4	38.2	31.4	33.8	35.1	28.2	32.2	32.2	31.1	26.1	
Bottom quintile	34.7	39.9	39.2	37.0	34.8	35.7	29.5	31.9	29.7	30.0	
**RII (95% CI)**	1.3 (1.1, 1.6)	1.5 (1.3, 1.7)	1.4 (1.1, 1.8)	1.4 (1.1, 1.6)	1.3 (1.1, 1.6)	1.2 (1.0, 1.5)	1.2 (1.0, 1.4)	1.2 (1.0, 1.5)	1.3 (1.1, 1.6)	1.1 (0.9, 1.4)	0.009
**SII (95% CI)**	0.1 (0.0, 0.2)	0.1 (0.1, 0.2)	0.1 (0.0, 0.2)	0.1 (0.0, 0.2)	0.1 (0.0, 0.2)	0.1 (0.0, 0.1)	0 (0.0, 0.1)	0.1 (0.0, 0.1)	0.1 (0.0, 0.1)	0 (0.0, 0.1)	0.025

## Discussion

Prevalence of all four behavioural risk-factors reduced over the course of the study period, although prevalence of insufficient fruit and vegetable consumption remained high. In terms of patterns by SEPs, for smoking and inadequate fruit and vegetable consumption, those in lower SEPs consistently had higher prevalence of the risk factors; for physical inactivity, this also became true by the end of the study period. Relative and absolute inequality grew over the period for physical inactivity and relative inequality but not absolute inequality grew for smoking. For fruit and vegetable consumption, the inequalities depended on SEPs measure: both absolute and relative inequality narrowed for women by neighbourhood deprivation and income, but for men both relative and absolute inequality widened by education. In contrast to other risk-factors, those in higher SEPs had higher prevalence of alcohol consumption above daily limits than those in the lower SEPs; this inequality was generally widening. In terms of co-occurrence of risk-factors, the picture was improving at a whole population level, with the prevalence of two or more risk-factors decreasing and the prevalence of no risk-factors increasing. However, those in lower SEPs had higher prevalence of two or more risk-factors and this inequality did not change significantly for any measure of SEPs, except for inequalities by income for women.

The inequalities in physical inactivity are concerning. Studies published around the start of time period noted that those with higher SEPs completed more leisure time physical activity than those with lower SEPs [20], with occupational physical activity higher in groups with lower SEPs. Examining total physical activity may have obscured differences in physical activity for leisure and non-leisure by SEPs. Future research should examine whether reductions in occupational and travel physical activity, particularly for low SEPs, have led to widening inequalities in total physical activity. A study of OECD countries using data collected up to 2014 noted that in England, Australia, Korea, Spain and the US, those with higher educational status had lower prevalence of insufficient physical activity, but the opposite was true in Chile and Mexico [21]. This suggests there may be a transition as countries have increasingly mechanised work and travel, in which risk of physical inactivity for those with low SEPs increases to a greater extent than for those with high SEPs. Following the COVID-19 pandemic, it is unlikely that England will ever return to having an economy in which occupational physical activity is accrued to any great extent by a large proportion of the population. Therefore, a focus on active travel and leisure time physical activity is needed. Increasing affordability of these may support increased physical activity for those with lower SEPs. Accessibility also needs to be considered, with neighbourhoods requiring investment to support active travel and recreation.

The persisting and/or widening inequalities in smoking behaviour requires attention. Tobacco use significantly increases the probability of dying prematurely as well as decreasing quality of life. Smoking has previously been identified as contributing the most to social inequalities in health outcomes [22]. Despite a number of population level policy interventions (ban on smoking in enclosed public spaces in 2007, ban on smoking in cars with people under 18 in 2015 and plain packaging in 2017), inequalities in smoking persist and have continued to widened for some of the SEPs, although tobacco use has decreased overall. Studies in other countries have similar persisting or widening socioeconomic inequalities in smoking behaviour [23, 24]. The most recent review to examine the inequality in impact of population tobacco control measures suggested that price increases and targeted population-level cessation support were the only interventions where there is consistent evidence of a greater effect among low SEPs smokers [25]. Re-visiting affordability of tobacco in England, and ensuring local authorities are able to maintain effective and accessible cessation services may support reducing inequalities in prevalence of tobacco use in the future. Social interventions may also be needed, as smoking behaviour spreads through social influences which may maintain higher smoking rates within social networks which share low SEP [26].

For alcohol, the pattern of higher levels of drinking in higher SEPs belies the fact that the greatest burden of alcohol-related harm falls on populations with lower SEPs [27]. Alcohol-related hospital admissions have increased over the time period, and this increase was more concentrated in deprived areas reflecting this paradox [28]. It is worth noting that we examined whether participants consumed more than a daily threshold of 3 units for women, 4 for men; but patterns of drinking more than a higher daily threshold such as heavy episodic drinking, or a weekly threshold might highlight other inequalities. There is evidence to suggest that low socioeconomic groups are more likely to drink at extreme levels, including four times the threshold [29], which this study did not examine. In a study of 17 European countries from 1980–2010, there was greater alcohol related mortality in those with lower educational status in all countries studied [30]. The study also found that relative educational inequality in alcohol related mortality increased over time in most countries and the absolute educational inequality in alcohol related harm increase markedly in Hungary, Lithuania, Estonia, Finland and Denmark, while staying stable in France, Switzerland, Spain and Italy [30].

Finally, the fruit and vegetable analysis clearly demonstrates that the measure of SEPs matters. Both relative and absolute inequalities are narrowing by neighbourhood deprivation and by income for women. Meanwhile, relative and absolute inequalities by education status are widening for men. Potentially, understanding which indicators of SEPs are associated with widening inequalities could suggest potential policy targets; in this case suggesting that a focus on diet for groups (particularly men) with poor education may be important. Meanwhile further narrowing of inequalities by neighbourhood deprivation and income might be supported by increasing access and affordability of fruit and vegetables. A study of the Scottish diet between 2001 and 2007 found very little change in absolute or relative inequalities in intakes of food or nutrients [31]. A study of OECD countries, analysing data collected between 2003 and 2013 or the closest available years, found that the largest relative and absolute educational inequalities were in Canada, England, Mexico and in Korean men and that trends in relative educational inequalities had increased or remained stable, while absolute educational inequalities had reduced or remained stable for men, while increasing for women (in contrast to our findings). The same study found that relative socio-economic inequalities (undefined in the report) had increased for men and decreased for women and absolute socio-economic inequalities had risen for both men and women [21]. None of these studies examined the same time period as our study, which may explain the differences in findings.

The strengths of this study are that we used robust, standardised national datasets with indicators that are comparable year on year and applied robust weighting for non-response. We were able to examine a range of SEPs measures and compare and contrast our findings. However, we used IMD 2015 for the whole study period, which may not be an accurate marker of deprivation across all the study years, and a around a fifth of the population had missing data for income which might have introduced bias in our findings.

Statistically, generalised linear models (log-Binomial regression) with logarithmic link function would have been the most appropriate method for our analyses, however the models repeatedly failed to converge in Stata when RII was close to 1. This is a known problem with log-Binomial regressions. We used generalised linear models (log-Gaussian regression) as suggested in the literature to address this issue [18].

Finally, we note that there are many measures and indicators that could have been chosen for each of the behavioural risk-factors studied, some of which are discussed above. For example, there are many dietary behaviours that are important for health, other than fruit and vegetable consumption and the threshold for examining the risk-behaviour could have been set differently (e.g. for physical inactivity we could have used < 150 min MVPA per week; for fruit and vegetable consumption we could have used < 1 portion per day). Furthermore the lack of consistent years data particularly on physical activity prevented us from exploring the co-occurrence of more than three risk-factors over the period.

Further research examining the trends in inequalities in prevalence of behavioural risk-factors for NCDs in other countries, which could be compared with our findings, could give additional insight into how the wider socio-political environment of England (and other countries) might be affecting inequalities in risk behaviours.

## Data Availability

Health Survey for England data are available to UK Academic institutions from the UK Data Archive subject to their end-user license. The reference for the data from each survey is listed below: 2003: National Centre for Social Research, “Health Survey for England, 2003. [data collection],” University College London, Department of Epidemiology and Public Health. 2nd Edition. UK Data Service. SN: 5098, 2010. http://doi.org/10.5255/UKDA-SN-5098-1 (accessed Nov. 09, 2021). 2004: National Centre for Social Research, “Health Survey for England, 2004. [data collection],” University College London, Department of Epidemiology and Public Health. 2nd Edition. UK Data Service. SN: 5439, 2010. http://doi.org/10.5255/UKDA-SN-5439-1 (accessed Nov. 09, 2021). 2005: National Centre for Social Research, “Health Survey for England, 2005. [data collection],” University College London, Department of Epidemiology and Public Health. 3rd Edition. UK Data Service. SN: 5675, 2011. http://doi.org/10.5255/UKDA-SN-5675-1 (accessed Nov. 09, 2021). 2006: National Centre for Social Research, “Health Survey for England, 2006. [data collection],” University College London, Department of Epidemiology and Public Health. 4th Edition. UK Data Service. SN: 5809, 2011. http://doi.org/10.5255/UKDA-SN-5809-1 (accessed Nov. 09, 2021). 2007: National Centre for Social Research, “Health Survey for England, 2007. [data collection],” University College London, Department of Epidemiology and Public Health. 2nd Edition. UK Data Service. SN: 6112, 2010. http://doi.org/10.5255/UKDA-SN-6112-1 (accessed Nov. 09, 2021). 2008: National Centre for Social Research, “Health Survey for England, 2008. [data collection],” University College London, Department of Epidemiology and Public Health. 4th Edition. UK Data Service. SN: 6397, 2013. http://doi.org/10.5255/UKDA-SN-6397-2 (accessed Nov. 09, 2021). 2009: National Centre for Social Research, “Health Survey for England, 2009. [data collection],” University College London, Department of Epidemiology and Public Health. 3rd Edition. UK Data Service. SN: 6732, 2015. http://doi.org/10.5255/UKDA-SN-6732-2 (accessed Nov. 09, 2021). 2010: NatCen Social Research, “Health Survey for England, 2010. [data collection],” Royal Free and University College Medical School. Department of Epidemiology and Public Health. 3rd Edition. UK Data Service. SN: 6986, 2015. http://dx.doi.org/10.5255/UKDA-SN-6986-3 (accessed Nov. 09, 2021). 2011: NatCen Social Research, “Health Survey for England, 2011. [data collection],” University College London, Department of Epidemiology and Public Health. UK Data Service. SN: 7260, 2013. http://dx.doi.org/10.5255/UKDA-SN-7260-1 (accessed Nov. 09, 2021). 2012: NatCen Social Research, “Health Survey for England, 2012. [data collection],” University College London, Department of Epidemiology and Public Health. UK Data Service. SN: 7480, 2014. http://dx.doi.org/10.5255/UKDA-SN-7480-1 (accessed Nov. 09, 2021). 2013: NatCen Social Research, “Health Survey for England, 2013. [data collection],” University College London, Department of Epidemiology and Public Health. UK Data Service. SN: 7649, 2015. http://dx.doi.org/10.5255/UKDA-SN-7649-1 (accessed Nov. 09, 2021). 2014: NatCen Social Research, “Health Survey for England, 2014. [data collection],” University College London, Department of Epidemiology and Public Health. 2nd Edition. UK Data Service. SN: 7919, 2016. http://doi.org/10.5255/UKDA-SN-7919-2 (accessed Nov. 09, 2021). 2015: NatCen Social Research, “Health Survey for England, 2015. [data collection],” University College London, Department of Epidemiology and Public Health. UK Data Service. SN: 8280, 2017. http://doi.org/10.5255/UKDA-SN-8280-1 (accessed Nov. 09, 2021). 2016: NatCen Social Research, “Health Survey for England, 2016. [data collection],” University College London, Department of Epidemiology and Public Health. 3rd Edition. UK Data Service. SN: 8334, 2019. http://doi.org/10.5255/UKDA-SN-8334-3 (accessed Nov. 09, 2021). 2017: University College London, “Health Survey for England, 2017. [data collection],” University College London, Department of Epidemiology and Public Health. 2nd Edition. UK Data Service. SN: 8488, 2021. http://doi.org/10.5255/UKDA-SN-8488-2 (accessed Nov. 09, 2021). 2018: National Centre for Social Research (NatCen), “Health Survey for England, 2018. [data collection],” University College London, Department of Epidemiology and Public Health. UK Data Service. SN: 8649, 2020. http://doi.org/10.5255/UKDA-SN-8649-1 (accessed Nov. 09, 2021). 2019: National Centre for Social Research (NatCen), “Health Survey for England, 2019. [data collection],” University College London, Department of Epidemiology and Public Health. UK Data Service. SN: 8860, 2020. http://doi.org/10.5255/UKDA-SN-8860-1 (accessed Aug. 20, 2022).
